# Circadian system functional status and sleep in blind subjects with and without conscious light perception

**DOI:** 10.3389/fphys.2026.1787735

**Published:** 2026-04-13

**Authors:** David Martínez-Martínez, Pedro González-Romero, Beatriz Rodríguez-Morilla, María Ángeles Bonmatí-Carrión, María Ángeles Rol, Pedro Francisco Almaida-Pagán

**Affiliations:** 1 Chronobiology and Sleep Laboratory, University of Murcia, Murcia, Spain; 2 IMIB-Arrixaca, Murcia, Spain; 3 Ciber Fragilidad y Envejecimiento Saludable (CIBERFES), Instituto de Salud Carlos III, Madrid, Spain; 4 Kronohealth SL, Murcia, Spain; 5 Department of Anatomy and Psychobiology, University of Murcia, Research Institute on Ageing (IUIE), Murcia, Spain

**Keywords:** blindness, circadian photoreception, daily habits, feeding times, light exposure, sleep

## Abstract

To understand circadian rhythms and sleep in an understudied population, which is particularly prone to suffer chronodisruption (CD), eighteen blind volunteers of 51.5 ± 3.6 years (Mean ± SEM) and 26 volunteers (51.8 ± 1.2) with no visual impairments wore the ambulatory circadian monitoring (ACM) device Kronowise^®^ for seven consecutive days in real-life conditions. Nine of the blind participants declared to have some sort of light perception while the other nine declared to lack conscious light perception. ACM combines measurements of distal skin temperature; motor activity, light exposure and feeding schedules, providing information about lifestyle and the bidirectional crosstalk between internal time and external synchronisers, which is paramount to determine a subject’s CD degree. We found a extraordinarily diverse population in terms of blindness aetiology and thus, in the degree of affectation of the participants visual and circadian systems. Our results pointed to poorer circadian health and sleep in the blind participants, which could be directly related to the impact of disease over circadian photoreception but also to disruption of daily habits. Compared to controls, blind participants showed significantly lower light exposure and physical activity values during the day and higher time of movement during the night. Besides, we analysed feeding schedules in the blind participants for the first time and found that their last meal of the day happened later than in controls, thus blind participants’ night fasting was shorter. Altogether, our results indicated substantial behavioural circadian alterations associated with the disease.

## 1 Introduction

Maintaining the different physiological processes in the appropriate temporal order is essential to keep health and well-being (Karatsoreos et al., 2011; Mazzoccoli et al., 2011). Circadian rhythm disruption or chronodisruption (CD) has been defined as a relevant disturbance of the internal temporal order of physiological, biochemical and behavioural circadian rhythms or in the regular phase relationship between internal circadian rhythms and exposure to environmental synchronisers (Erren et al., 2003; Garaulet and Madrid, 2010), being light/dark cycle the most important. CD connects light, biological rhythms and the development of several life-threatening diseases (Erren et al., 2009, 2003). In 2007 (revised in 2019), the International Agency for Research on Cancer (WHO) classified ‘shift work that involves circadian disruption’ as potentially carcinogenic in humans (Straif et al., 2007; International Agency for Research on Cancer (WHO), 2020), and recently, the most prestigious scientific journals, Nature, Science and Current Biology have claimed about health risks produced by an inadequate light exposure, which has also been related to a higher probability to suffer cardiovascular disease, cognitive impairment, affective disorders, sleep alterations, inflammatory and metabolic disorders and accelerated ageing (Czeisler, 2013; Kantermann, 2013; Mehta and Zhu, 2009; Verma et al., 2023).

Blindness is a visual impairment (VI) affecting 0.7% of the world population and its incidence is expected to grow in the next years due to the ageing of population and the increasing prevalence of disorders such as diabetes (Gómez-Ulla de Irazazábal and Odategui-Parra, 2012). The most recent statistics speak of around 1 million people with VI in Spain, of which 70,490 are blind, this is around 1.5 blind persons per 1,000 inhabitants (to be considered as blind in Spain, a person must be affected in both eyes by at least one of two visual requirements: 1) visual acuity equal or below 0.1, obtained with the best possible optic correction, and/or 2) visual field reduced to 10 degrees or under) (Record of ONCE affiliates, December 2022, https://www.once.es/dejanos-ayudarte/afiliacion/datos-de-afiliados-a-la-once).

Most people with VI, at higher levels, are particularly prone to CD due to their inability to synchronise their circadian clock with the environmental light-dark cycle (Medeiros da Silva et al., 2016; Warman et al., 2011). The International Classification of Sleep Disorders (ICSD-3) of the American Academy of Sleep Medicine (https://aasm.org/) attests to that fact, saying that more than half of totally blind individuals have non-standard 24-hour circadian rhythms and 50% to 80% of blind people present sleep disorders (American Academy of Sleep Medicine, 2014). The situation worsens even more when we consider the difficulties of access to reference health services and, therefore, the delay in the diagnosis of disorders (Medeiros da Silva et al., 2016).

Since the early 2000s, we know the photoreceptors and pathways driving light into the central pacemaker of the circadian system (the suprachiasmatic nuclei) and entraining the circadian rhythms (Berson et al., 2002). While image forming is primarily driven by rods and cones from the outer retina, whose signal are transmitted to the vision-processing centre of the brain, there is a third sort of photoreceptor, a type of ganglion cells located in the inner retina called intrinsically photosensitive retinal ganglion cells (ipRGC), which contain melanopsin (Davies et al., 2012; Hankins et al., 2008; Vandewalle et al., 2007), a specialised photopigment that is especially sensitive to light in the 460-480nm range (Canteras et al., 2011; Kawasaki and Kardon, 2007; Morin et al., 2006). ipRGC’s axons converge into the retino-hypothalamic tract and lead to the central pacemaker (Grone et al., 2011; Maywood et al., 2007; Moore and Speh, 2004), where they make synaptic contact with clock neurons. Although it has been suggested that ipRGCs contribute to basic vision (e.g. low-spatial frequency contrast, brightness detection, and adjustment of the sensitivities of rods and cones) in humans and rodents (Vandewalle et al., 2018), their main role would be transmitting light information to the circadian system. We now know that, among the blind population, there are people who retain non-image-forming photoreception, presumably via the ipRGC (Medeiros da Silva et al., 2016), offering a unique opportunity to investigate light impacts in the absence of conscious vision.

In a survey conducted with 1,073 blind people (53% with no light perception), sleep patterns were altered compared to non-blind people (Leger et al., 1996) and in a comparative study, among people with no light perception, 83% of them had at least one sleep problem, such as difficulty of sleeping, night-time awakenings, decreased sleep duration and daytime sleepiness, compared with 57% in the control group (Tabandeh et al., 1998). Therefore, these investigations point to the fact that the higher the degree of severity of VI, the greater the sleep disorders, and the prevalence of sleep ailments is higher in blind people with no light perception compared to blind people with some sort of light perception and control (people without any VI) [(Adeoti, 2010; Das et al., 2006; Vervloed et al., 2003; Warman et al., 2011) in (Medeiros da Silva et al., 2016)].

The overall aim of this study was to evaluate the status of the circadian system (CS) and sleep in a Spanish blind population in real life, in comparison with a control VI free population. Blind subjects with some sort of conscious light perception (CLP) and blind participants with NO CLP were separately analyzed to check for possible differences. With that purpose, multivariable ambulatory circadian monitoring or ACM, through wearable technology, was used, as it provides objective information about lifestyle and the bidirectional crosstalk between internal time and external synchronisers, which is paramount to determine a subject´s propensity to suffer CD (Ortiz-Tudela et al., 2014a).

## 2 Material and methods

### 2.1 Participants

Eighteen volunteers (28% female) of 51.5 ± 3.6 years of age (Mean ± SEM) (**Table 1**) from Murcia’s Region were recruited between May and June 2023 through the Spanish National Organization of the Blind (ONCE). All participants were free from any significant health condition other than blindness, and they had not crossed across time zones in the last month. Although they could not see any images, nine of them (11% females) declared to have some sort of light perception (CLP) while the other nine (44% females) declared to lack conscious light perception (NO CLP) (**Table 1**). As controls, we recruited (between May and June 2024) 26 volunteers (50% female) with no visual impairments and with similar age and body mass index than blind participants (**Table 1**). Volunteers were encouraged to maintain their normal lifestyle during the week of the study. All participants received appropriate information about the research characteristics and signed an informed consent form prior to their inclusion in accordance with the Declaration of Helsinki. This work was approved by the Ethics Committee of University of Murcia (ID 2108/2018). Data from the volunteers were included in a database and were protected according to Spanish Law 15/1999 of 13 September.

**Table 1:** Characteristics of blind participants (total blind population, with conscious light perception [CLP] and with no conscious light perception [NO CLP]) and controls.

	N	Age (years)	BMI
Control	26 (50% female)	51.8 ± 1.2	25.5 ± 0.7
Blind	18 (28% female)	51.5 ± 3.6	26.6 ± 1.1
CLP	9 (11% female)	51.3 ± 4.6	27.3 ± 1.9
Causes			
Retinitis pigmentosa	3		
Retinopathy of prematurity	2		
Glaucoma	1		
Brain tumour	1		
Side effects of penicilin	1		
High myopia	1		
NO CLP	9 (44% female)	51.7 ± 5.9	25.9 ± 1.1
Causes			
Traffic accident	4		
Retinopathy of prematurity	3		
Glaucoma	1		
Side effects of anaesthesia	1		

Age and body mass index (BMI) data expressed as mean ± SEM.

### 2.2 Ambulatory circadian monitoring for the analysis of circadian system status and inferred sleep

An ACM device the size of a wrist watch (Kronowise^®^ 3.0, Kronohealth S.L., Spain) and equipped with a temperature sensor, a MEMS calibrated triaxial accelerometer and three light sensors [(the technical characteristics of the ACM Kronowise^®^ 3.0 device have been previously described by Madrid-Navarro et al. (2018)] was placed on the non-dominant hand over the course of seven consecutive days and participants were instructed to remove the device only to shower. The main variables recorded by this device were wrist skin temperature (WT), triaxial acceleration (A), time in movement (TM), triaxial wrist position (P), and exposure to light on three spectral bands (visible, blue light with a wavelength of 460–490 nm and infrared light >700 nm). The sampling frequencies per period (epoch= 30 sec.) were: 10Hz (300 readings per epoch) for the A and TM measurements, 1Hz (30 reading per epoch) for WT and light exposure and 0.033Hz (1 reading per epoch) for P and event marker, capturing ∼23,000,000 data points over the course of a week.

Data were analysed by the chronobiological software Circadianware, implemented in the online Kronowizard platform (https://kronowizard.um.es/).

The following were selected from a total of 14 variables recorded by Kronowise^®^:

a. Wrist skin temperature (WT) as a variable with a strong endogenous circadian component and representative of autonomic balance at the skin vessel level. WT rises at night, preceding sleep, and is correlated and phase-advanced with respect to the night decrement of central body temperature (CBT), suggesting that heat loss from the extremities may drive the CBT circadian rhythm (Raymann et al., 2005; Sarabia et al., 2008; Van Someren, 2003).
b. Tilt of the x-axis (P, radius and ulna perpendicular axis), which oscillates between 0° for maximum horizontality and ±90° for maximum verticality. This variable allows to identify posture changes during immobility periods, so as the amplitude of movements, from the difference of grades between one sample and the previous. The decrease in P variability allows to identify the beginning of sleep periods.
c. Acceleration or intensity of movement (A). It was calculated as the sum (absolute value) of the acceleration vector values (in g, Proportional Integral Mode algorithm) recorded in the 300 samplings (10 Hz) of one epoch (30 sec.). To facilitate the comparison with other devices, the sum of acceleration vector values per epoch was divided by the sampling frequency (10 Hz) and thus, A data were expressed as g/30 sec.

A indicates speed of movement and force, but not the duration or frequency of the movement. A was categorized considering the sum of the acceleration of the three axes in each epoch with the following classes: vigorous (≥100), moderate (>50), light (>25), sedentary (>2), and rest (≤ 2).

d. Time in movement (TM) was calculated as the sum of samples per epoch (second tenths) in which activity on any of the three axes of the accelerometer overcame a threshold of 0.05 g (Time Above Threshold algorithm) from the 300 samplings (10 Hz) recorded in 1 epoch (30 sec.). TM data can be easily transformed from sec. tenths per 30 sec. to sec. per min. TM is particularly useful to discriminate between sleep and wake states and together with A offers information on a circadian variable more dependent on willingness.
e. Three light sensors on the front to record visible (LE), infrared and blue light (BLE), with a range of between 0.01 and 43,000 lux, with internal self-adjustment according to the level of luminance and suppression of flicker at 50/60Hz. For this study, only LE was used to calculate the integrated variable TAPL (Madrid-Navarro et al., 2019).

For light source categorization (indoors, outdoors), the proportion of infrared versus full light spectrum (IR/Full ratio) was calculated from data recorded by ACM light sensors and then by applying an algorithm previously developed by our group, which correlates ACM data with spectroradiometer measures (Arguelles-Prieto et al. (2019).

### 2.3 Automatic inference of sleep and wake states.

To automatically infer sleep and wake periods, a two-phase procedure, previously described by Madrid-Navarro et al. (2019), was used. First, sleep and wake periods were automatically inferenced using the TAPL algorithm, a modification of the previously published TAP algorithm, which integrated temperature (T), activity (A) and position (P) (Ortiz-Tudela et al., 2010) to additionally integrate exposure to LE, implemented on the Kronowizard website. TAPL can be used to express general activation, where a value of 0 was an indicator of deep rest, characterized by immobility, vasodilation of the skin and low levels or absence of LE (probable sleep), while 1 corresponded to a wake state. A time period was classified as sleep when the TAPL value fell beneath a pre-set threshold, previously validated by PSG (Ortiz-Tudela et al., 2014a). Once the main inferred sleep and wake periods had been detected by means of TAPL, we proceeded to mark the probable intra-sleep awakening episodes to improve the precision of the estimates, as previously validated through comparison with PSG as the standard (Ortiz-Tudela et al., 2014a). The Keywake^®^ algorithm was used to mark these periods, using artificial intelligence and based on the time in movement from the 4 min before and 2 min after each time period evaluated (Madrid-Navarro et al., 2019). All these calculations are implemented on the Kronowizard platform.

### 2.4 Calculation of circadian parameters

To analyse any possible differences among circadian patterns between blind and control subjects, a non-parametric analysis was carried out as described in the literature (Refinetti et al., 2007), which includes the following indexes: 1) Interdaily Stability (IS), which quantifies the invariability of the rhythm between days; 2) Intradaily Variability (IV), which gives an indication of the fragmentation of the rhythm and 3) Relative Amplitude (rhythm amplitude, RA) (Witting et al., 1990). For those variables whose acrophase occurred during the daytime (LE, BLE, A, TM and TAPL), RA was calculated as the difference between M10 (average for the 10 consecutive hours with the maximum values, measured in 10-min intervals) and L5 (average for the 5 consecutive hours with the minimum values, measured in 10-min intervals), divided by the sum of M10 and L5, as previously reported by Witting et al. (1990) for activity. However, since WT and sleep acrophases occur during the rest period, RA was calculated as the difference between M5 (average for the 5 consecutive hours with the maximum values, measured in 10-min intervals) and L10 (average for the 10 consecutive hours with the minimum values, measured in 10-min intervals), divided by their sum (Martinez-Nicolas et al., 2019). To normalize relative amplitude among variables (NRA), the highest and the lowest 5% values were recoded as 1 and 0, respectively, and the values in between were rescaled for the new range. The night phase markers were obtained using the timing for L5 (LE, BLE, A, TM and TAPL) or M5 (WT and sleep), while the timing of L10 (WT and sleep) or M10 (LE, BLE, A, TM and TAPL) was used as day phase markers, as previously described (Martinez-Nicolas et al., 2011).

The Environmental Synchronization (ES) parameter was calculated to characterize the level of synchronization between the endogenous phase marker of one circadian variable during the resting period (M5T or L5T) and the midpoint of natural darkness (Mnd), taking as reference the official time in the location where the recording is made (i.e. 2 am for Spain, considering standard or daylight-saving time when appropriate) (Vicente-Martinez et al., 2023):


$$ES=1\;-\left(\frac{\left|M5T\;or\;L5T-M_{nd}\right|}{12}\right)$$

Since the maximum possible desynchronization for one rhythm regarding environmental darkness is 12 h, values are normalized respecting this maximum value. Therefore, an ES = 1 occurs when the midpoint of sleep coincides with the natural darkness, while an ES = 0 would happen when the midpoint of sleep takes place at 2 pm.

The Internal Synchronization (IntS) was used as an indicator of the coincidence between the circadian phases of different circadian rhythms. IntS was calculated as the difference in phase (in hours) between the timing of wrist temperature M5 and the timing of time in movement L5, used as night phase markers, divided by 12h, which is the maximum difference between the two variables (Vicente-Martinez et al., 2023):


$$IntS=1-\left(\frac{\left|WT\;TM5-TM\;TL5\right|}{12}\right)$$

This way, a subject with a timing of WT M5 at 3am and a timing of TM L5 at 6am will have an IntS= 1- (|3-6|/12) = 0.75. IntS shifts closer to 1 as the synchronization among the rhythms increases, and an IntS equal to 0 means that the difference between the two variable phases is 12.

Finally, two global indexes of the robustness of the circadian system were calculated. First, the A/T index was calculated as the ratio Acceleration M10/Time in movement L5 (Madrid-Navarro et al., 2018). If we consider A or acceleration of physical activity during the day as an indicator of physical vigour and the TM during the night as a marker of the fragmentation of the sleep rhythm, the relationship between the two parameters makes it possible to determine the degree of contrast between day and night and can also be used as an indicator of biological ageing. In particular, low values in this index reflect low levels of A during wakefulness together with high values of TM during sleep (lower sleep depth), indicating ageing and circadian frailty. Second, the Circadian Health Status (CHS), which integrates three main characteristics of a healthy rhythm: NRA, IS, and ES (Vicente-Martinez et al., 2023). CHS was calculated as:


$$CHS=\;\frac{IS+NRA+ES}{3}$$

CHS values range from 0 to 1 where 1 means that a rhythm is perfectly regular, with a high amplitude and centred in the natural darkness.

### 2.5 Calculation of sleep parameters

The sleep and wake periods were automatically inferred as previously described; however, the precise moments at which the subject went to bed and got up were recorded manually as there was a great variability of habits among participants, so that the calculation of sleep parameters was circumscribed to the periods manually marked as bed periods. Bedtime was set after following a drop in activity level and when visible light went off. Get up time was defined using the following indicators: increase in activity level, decrease in WT and increase in light level above 1.0 μW/cm^2^. The rest of the sleep parameters were calculated automatically, as described by Madrid-Navarro et al. (2019).

### 2.6 Night fasting

Volunteers were asked to indicate when they eat by pressing for five seconds the event marker button in the Kronowise^®^ at the beginning of each meal. Valid recordings from 9 blind participants (51.6 ± 4.8 years, 11% females) and 20 controls (52.0 ± 1.5 years, 55% females) were gathered. Night fasting length, phase, regularity and relationship with sleep were calculated. Particularly, regularity was assessed with Composite Phase Deviation, as described by Fischer et al. (2016).

### 2.7 Statistical analysis

While the control sample was constituted by 50% females, representing the general population, the blind sample only counted on 28% females, this gender being underrepresented regarding the general blind population. Considering that it is difficult to access total blind subjects and to have control over gender proportions, we performed a two-way ANOVA to check for possible interactions between ‘group’ (blind participants *versus* controls, CLP or NO CLP) and ‘gender’ variables for every parameter and index. As only significant results for WT NPIs between the ‘Blind’ and ‘Control’ populations were found, the remaining mean values of the circadian and sleep parameters calculated by ACM for blind participants *versus* controls, and CLP *versus* NO CLP were compared using an independent-samples Student’s t-test after checking for data normality (Kolmogorov-Smirnov test). Due to the high number of tests, *p* values were corrected using the Benjamini-Hochber method, in order to reduce the False Discovery Rate (**Supplementary Table 1**) (Haynes, 2013). Only adjusted *p* values are shown in text and those under 0.05 were considered statistically significant. All statistical analyses were performed with IBM SPSS Statistics version 28.0.1.1 (SPSS, Inc., Chicago, IL, United States). All data are expressed as mean ± SEM and were processed using Microsoft Office Excel 2019.

## 3 Results

### 3.1 Causes of blindness

The aetiology of blindness among volunteers was very diverse (**Table 1**), finding up to eight different causes in a sample of eighteen participants. Retinopathy of prematurity was the most frequent (5 cases), followed by traffic accidents causing ocular mass loss and/or retinal detachment (4 cases), retinitis pigmentosa (3 cases), glaucoma (2 cases), high myopia (1), brain tumour (1) and side effects of anaesthesia (1) and penicillin (1).

Except for the cases affected by bilateral enucleation (2), it was found that individuals diagnosed with the same ocular disease declared different visual phenotypes (i.e. keeping or lacking CLP).

### 3.2 Gender interactions

**Figure 1:**
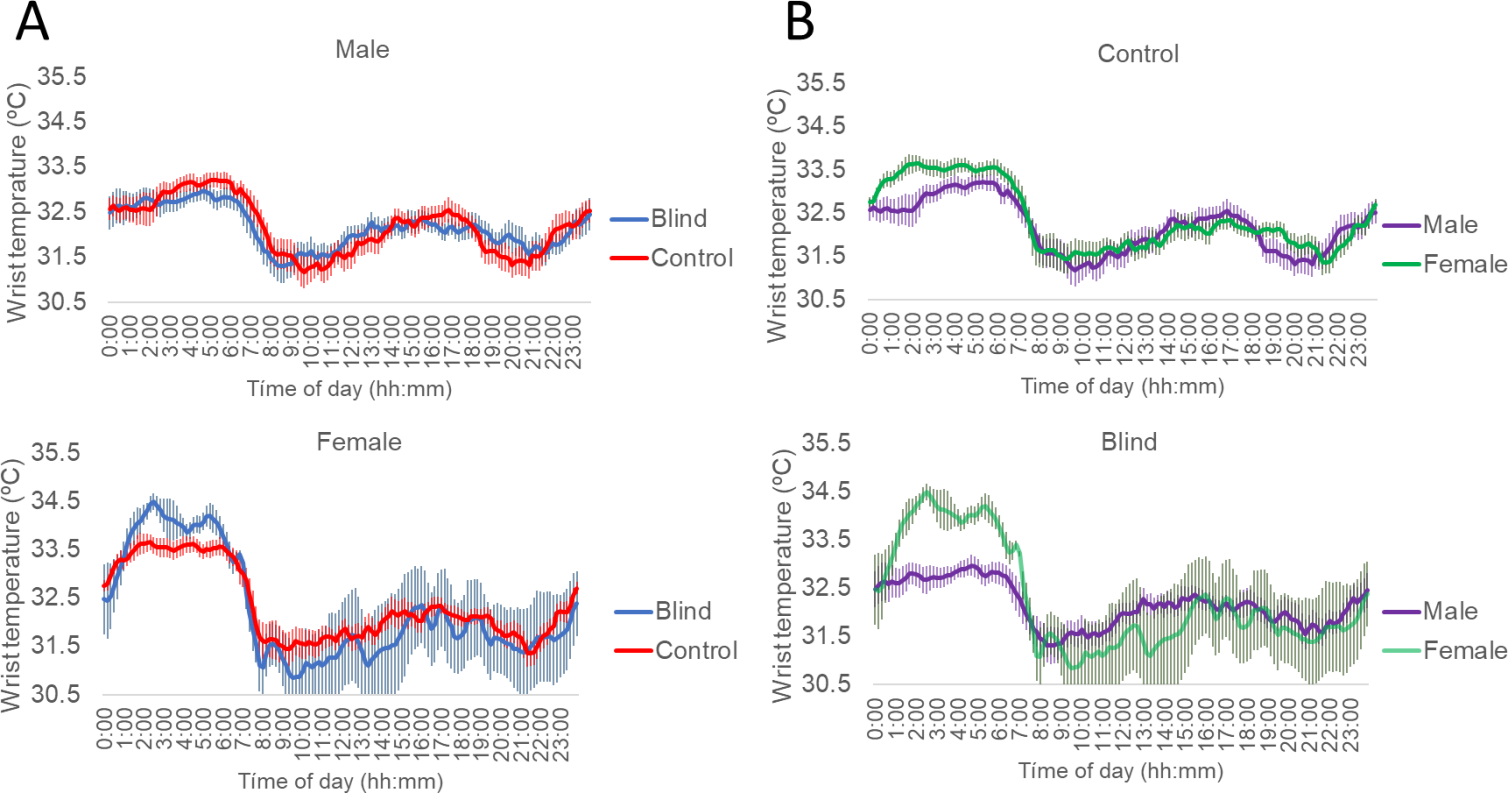
Mean-waveforms of wrist temperature for **(A)** total blind (blue line) and control subjects (red line) separated by gender and **(B)** male (purple line) and female participants (green line) separated by group. n for blind male: 13, blind female: 5, control male: 13 and control female: 13. Data are presented as the mean ± SEM (vertical bars).

Statistically significant interactions between group and gender were only found for the wrist temperature rhythm (WT), particularly for maximum night values (M5, *F[1, 40] = 8.374*, *p = 0.030*), regularity (IS, *F[1, 40]= 6.980, p= 0.030*), normalized amplitude (NRA, *F[1, 40]= 6.418, p= 0.030*) and circadian health score (CHS, *F[1, 40]=* 6.483,*p= 0.030*) (**Tables 2** and **3**). In general, females showed higher WT CHS (this index integrates IS, NRA and environmental synchronization) than males, this pointing to better WT rhythms in females. These differences were particularly significant in blind women compared to males (**Figure 1**).

**Table 2:** Non-parametrical indexes of circadian rhythms corresponding to the blind participants (n=18) or their respective controls (n=26).

	TAPL		Sleep		LE		BLE		WT		A		TM	
	Blind	Control	Blind	Control	Blind	Control	Blind	Control	Blind	Control	Blind	Control	Blind	Control
M5	–	–	**0.82 ± 0.02**	**0.89 ± 0.01**	–	–	–	–	33.3 ± 0.2	33.4 ± 0.1^¥^	–	–	–	–
M5T	–	–	3.68 ± 0.31	3.94 ± 0.18	–	–	–	–	5.25 ± 1.10	4.17 ± 0.58	–	–	–	–
L10	–	–	0.01 ± 0.01	0.02 ± 0.00	–	–	–	–	31.6 ± 0.2	31.7 ± 0.15	–	–	–	–
L10T	–	–	14.39 ± 0.52	14.22 ± 0.32	–	–	–	–	13.6 ± 1.1	14.4 ± 0.7	–	–	–	–
M10	0.49 ± 0.01	0.52 ± 0.01	–	–	**1.50 ± 0.13**	**1.89 ± 0.05**	1.13 ± 0.10	1.39 ± 0.05	–	–	**15.4 ± 1.0**	**20.2 ± 1.1**	29.6 ± 1.4	30.1 ± 1.0
M10T	15.06 ± 0.65	14.92 ± 0.32	–	–	14.55 ± 0.31	14.53 ± 0.16	14.38 ± 0.26	14.52 ± 0.16	–	–	15.5 ± 0.6	15.4 ± 0.4	14.9 ± 0.6	15.2 ± 0.3
L5	0.06 ± 0.01	0.05 ± 0.00	–	–	0.01 ± 0.01	0.01 ± 0.00	0.00 ± 0.00	0.00 ± 0.00	–	–	2.36 ± 0.14	2.11 ± 0.10	2.02 ± 0.30	1.32 ± 0.13
L5T	3.89 ± 0.29	3.85 ± 0.17	–	–	3.27 ± 0.30	3.62 ± 0.18	3.14 ± 0.28	3.43 ± 0.21	–	–	3.80 ± 0.29	3.87 ± 0.18	3.83 ± 0.30	3.84 ± 0.16
IS	0.48 ± 0.02	0.52 ± 0.01	0.65 ± 0.03	0.72 ± 0.01	0.54 ± 0.04	0.62 ± 0.02	0.49 ± 0.03	0.56 ± 0.02	0.39 ± 0.04	0.43 ± 0.02^¥^	0.32 ± 0.02	0.33 ± 0.01	0.45 ± 0.02	0.45 ± 0.01
IV	0.04 ± 0.00	0.03 ± 0.00	0.30 ± 0.02	0.28 ± 0.01	0.07 ± 0.01	0.07 ± 0.00	0.08 ± 0.01	0.09 ± 0.00	0.0 ± 0.0	0.0 ± 0.0	0.37 ± 0.02	0.33 ± 0.01	0.25 ± 0.01	0.25 ± 0.01
NRA	**0.43 ± 0.01**	**0.48 ± 0.01**	**0.81 ± 0.02**	**0.88 ± 0.01**	**0.50 ± 0.04**	**0.63 ± 0.02**	1.00 ± 0.00	1.00 ± 0.00	0.35 ± 0.05	0.35 ± 0.03^¥^	**0.37 ± 0.03**	**0.52 ± 0.03**	0.69 ± 0.04	0.72 ± 0.03
ES	0.83 ± 0.02	0.84 ± 0.01	0.85 ± 0.02	0.84 ± 0.01	0.87 ± 0.02	0.86 ± 0.01	0.88 ± 0.02	0.87 ± 0.01	0.74 ± 0.07	0.81 ± 0.03	0.84 ± 0.02	0.84 ± 0.01	0.83 ± 0.02	0.84 ± 0.01
CHS	**0.67 ± 0.01**	**0.70 ± 0.01**	0.78 ± 0.02	0.83 ± 0.01	0.70 ± 0.02	0.76 ± 0.01	0.58 ± 0.02	0.63 ± 0.01	0.43 ± 0.04	0.46 ± 0.02^¥^	**0.48 ± 0.01**	**0.54 ± 0.01**	0.58 ± 0.02	0.63 ± 0.01

Main characteristics of the circadian rhythms studied: TAPL (arbitrary units, 0–1), sleep probability, light exposure (LE, log_10_ lux), blue light exposure (BLE, log_10_ lux), distal skin temperature (WT, °C); acceleration of movement (A, g/30 sec); and time in movement (TM, sec/min).

The values are expressed as the mean ± SEM. M5 and M5T, mean value and mid-point time of the 5 consecutive hours with the highest values for WT and sleep probability; L10 and L10T, mean value and mid-point time of the 10 consecutive hours with the lowest values for WT and sleep probability; M10 and M10T, mean value and mid-point time of the 10 consecutive hours with the highest values for TAPL, LE, BLE, A and TM; L5 and L5T, mean value and mid-point time of the 5 consecutive hours with the lowest values for TAPL, LE, BLE, A and TM; BLE, blue light exposure; IS, interdaily stability; IV, intradaily variability; LE, visible light exposure; NRA, normalized relative amplitude; ES, environmental synchronization; CHS, circadian health status. P-values were corrected for multiple testing with the Benjamini-Hochberg method. A student’s t-test was used to compare means from the blind participants and their controls for each variable and parameter Significant differences (p-adjusted<0.05) are shown in bold type. A two-way ANOVA was conducted to analyse possible interactions between group (blind or control) and gender. ‘¥’ symbols indicate that this interaction is significant (p-adjusted<0.05) for that variable and parameter.

**Table 3:** Main sleep parameters from total blind participants (n=18) and controls (n=26).

	Blind	Control
Sleep latency, SL (min)	14.6 ± 2.1	11.9 ± 0.7
Sleep Interval, SI	414.0 ± 14.7	422.0 ± 8.0
WASO (min)	54.0 ± 5.8	43.9 ± 3.0
Sleep efficiency, SE (%)	**81.6 ± 1.4**	**85.9 ± 0.7**
Awakenings (n°/h)	2.8 ± 0.2	3.1 ± 0.2
Total time of movement, TTM (min)	9.0 ± 1.1	9.0 ± 0.9
Activity 2h before SO (°)	9.4 ± 0.8	9.6 ± 0.6
Activity 2h after waking (°)	**14.5 ± 1.2**	**20.5 ± 1.1**
A/T index	**11.1 ± 1.5**	**18.6 ± 2.0**
Internal Synchronization (IntS)	0.89 ± 0.09	0.97 ± 0.05
WT during sleep (°C)	33.3 ± 0.2	33.4 ± 0.1
WT 2h after waking (°C)	31.4 ± 0.3	31.4 ± 0.2
Visible light during sleep (log lux)	0.03 ± 0.01	0.02 ± 0.01
Blue light during sleep (log lux)	0.02 ± 0.01	0.01 ± 0.00
Visible light 2h before SO (log lux)	0.46 ± 0.09	0.69 ± 0.05
Blue light 2h before SO (log lux)	0.14 ± 0.04	0.15 ± 0.03
Visible light 2h after waking (log lux)	**1.06 ± 0.11**	**1.71 ± 0.07**
Blue light 2h before SO (log lux)	**0.61 ± 0.07**	**0.90 ± 0.06**
Napping time (min)	20.2 ± 11.2	13.2 ± 2.6

Data expressed as mean ± SEM. AT, awakening time (last time period marked as sleep before the get up time); SE, percentage of time asleep with regard to the time in bed; SL, total time in minutes between bed time and SO; SO, sleep onset (first time period marked as sleep after bed time); TIB, period of time between bed time and get up time; TST, time marked as sleep during the sleep interval; TTM, total minutes in the sleep interval during which movement has been detected; WASO, wake after sleep onset time (total minutes marked as awake after SO); WT, wrist skin temperature. A student’s t-test was used to compare Blind participants, and Control means for each parameter. P-values were corrected for multiple testing with the Benjamini-Hochberg method. Significant differences (p-adjusted<0.05) are shown in bold type. A two-way ANOVA was conducted to analyse possible interactions between group (blind or control) and gender. No significant differences were found.

When total blind participants were divided in two groups: those with conscious light perception (CLP) and those without conscious light perception (NO CLP), no statistically significant interactions between ‘group’ and ‘gender’ were found for any non-parametric index or sleep parameter (**Tables 4** and **5**).

**Table 4:** Non-parametrical indexes of circadian rhythms corresponding to the blind participants with conscious light perception (CLP, n=9) and the blind subjects with no conscious light perception (NO CLP, n=9).

	TAPL		Sleep		LE		BLE		WT		A		TM	
	CLP	NO CLP	CLP	NO CLP	CLP	NO CLP	CLP	NO CLP	CLP	NO CLP	CLP	NO CLP	CLP	NO CLP
M5	–	–	0.81 ± 0.03	0.83 ± 0.03	–	–	–	–	33.3 ± 0.2	33.4 ± 0.3	–	–	–	–
M5T	–	–	3.80 ± 0.50	3.56 ± 0.39	–	–	–	–	3.03 ± 0.54	7.47 ± 1.90	–	–	–	–
L10	–	–	0.01 ± 0.00	0.02 ± 0.01	–	–	–	–	31.7 ± 0.2	31.5 ± 0.5	–	–	–	–
L10T	–	–	14.49 ± 0.83	14.30 ± 0.66	–	–	–	–	14.4 ± 1.5	12.7 ± 1.6	–	–	–	–
M10	0.50 ± 0.02	0.49 ± 0.02	–	–	1.58 ± 0.20	1.42 ± 0.17	1.19 ± 0.169	1.06 ± 0.14	–	–	16.7 ± 1.7	14.2 ± 1.1	31.0 ± 2.0	28.2 ± 2.0
M10T	14.37 ± 0.92	15.74 ± 0.91	–	–	14.71 ± 0.57	14.39 ± 0.30	14.49 ± 0.47	14.28 ± 0.24	–	–	15.1 ± 1.0	15.8 ± 0.8	14.5 ± 0.9	15.2 ± 0.8
L5	0.06 ± 0.01	0.06 ± 0.01	–	–	0.01 ± 0.00	0.02 ± 0.01	0.00 ± 0.00	0.00 ± 0.00	–	–	2.40 ± 0.13	2.31 ± 0.27	1.95 ± 0.28	2.09 ± 0.55
L5T	4.14 ± 0.40	3.64 ± 0.44	–	–	3.51 ± 0.47	3.04 ± 0.40	3.39 ± 0.39	2.88 ± 0.41	–	–	4.00 ± 0.40	3.60 ± 0.43	3.96 ± 0.43	3.71 ± 0.44
IS	0.48 ± 0.02	0.47 ± 0.02	0.64 ± 0.04	0.66 ± 0.04	0.52 ± 0.06	0.55 ± 0.05	0.48 ± 0.06	0.50 ± 0.04	0.39 ± 0.04	0.39 ± 0.07	0.33 ± 0.02	0.32 ± 0.02	0.46 ± 0.03	0.44 ± 0.03
IV	0.03 ± 0.00	0.04 ± 0.00	0.34 ± 0.03	0.25 ± 0.02	0.08 ± 0.01	0.07 ± 0.01	0.09 ± 0.02	0.08 ± 0.01	0.0 ± 0.0	0.0 ± 0.0	0.39 ± 0.02	0.34 ± 0.03	0.25 ± 0.02	0.25 ± 0.02
NRA	0.44 ± 0.02	0.42 ± 0.02	0.80 ± 0.03	0.81 ± 0.03	0.52 ± 0.07	0.47 ± 0.06	0.99 ± 0.00	1.00 ± 0.00	0.33 ± 0.04	0.37 ± 0.10	0.41 ± 0.05	0.34 ± 0.03	0.73 ± 0.05	0.65 ± 0.05
ES	0.82 ± 0.03	0.84 ± 0.02	0.85 ± 0.04	0.85 ± 0.02	0.86 ± 0.03	0.89 ± 0.02	0.87 ± 0.03	0.89 ± 0.02	0.87 ± 0.03	0.61 ± 0.13	0.83 ± 0.03	0.84 ± 0.02	0.84 ± 0.04	0.83 ± 0.02
CHS	0.67 ± 0.01	0.67 ± 0.01	0.77 ± 0.03	0.79 ± 0.03	0.71 ± 0.03	0.69 ± 0.03	0.58 ± 0.03	0.58 ± 0.03	0.44 ± 0.04	0.41 ± 0.08	0.49 ± 0.02	0.48 ± 0.02	0.58 ± 0.04	0.58 ± 0.03

Main characteristics of the circadian rhythms studied: TAPL (arbitrary units, 0–1), sleep probability, light exposure (LE, log_10_ lux), blue light exposure (BLE, log_10_ lux), distal skin temperature (WT, °C); acceleration of movement (A, g/30 sec); and time in movement (TM, sec/min).

The values are expressed as the mean ± SEM. M5 and M5T, mean value and mid-point time of the 5 consecutive hours with the highest values for WT and sleep probability; L10 and L10T, mean value and mid-point time of the 10 consecutive hours with the lowest values for WT and sleep probability; M10 and M10T, mean value and mid-point time of the 10 consecutive hours with the highest values for TAPL, LE, BLE, A and TM; L5 and L5T, mean value and mid-point time of the 5 consecutive hours with the lowest values for TAPL, LE, BLE, A and TM; BLE, blue light exposure; IS, interdaily stability; IV, intradaily variability; LE, visible light exposure; NRA, normalized relative amplitude; ES, environmental synchronization; CHS, circadian health status. P-values were corrected for multiple testing with the Benjamini-Hochberg method. A student’s t-test was used to compare means from CLP and NO CLP participants for each variable and parameter. No significant differences were found. A two-way ANOVA was conducted to analyse possible interactions between group (CLP or NO CLP) and gender. No significant differences were found.

**Table 5:** Main sleep parameters from blind participants with conscious light perception (CLP, n=9) and blind subjects with no CLP (n=9).

	CLP	NO CLP
Sleep latency, SL (min)	13.8 ± 3.6	16.2 ± 2.6
Sleep Interval, SI	403.4 ± 17.2	430.8 ± 25.1
WASO (min)	58.4 ± 9.2	52.2 ± 8.0
Sleep efficiency, SE (%)	80.4 ± 2.6	82.2 ± 1.5
Awakenings (n°/h)	3.3 ± 0.3	2.3 ± 0.2
Activity 2h before SO (°)	9.1 ± 1.1	10.1 ± 1.1
Activity 2h after waking (°)	16.6 ± 2.1	13.1 ± 1.1
A/T index	10.3 ± 1.7	10.8 ± 2.5
Internal Synchronization (IntS)	1.08 ± 0.04	0.69 ± 0.16
WT during sleep (°C)	33.4 ± 0.2	33.3 ± 0.3
WT 2h after waking (°C)	31.6 ± 0.3	31.2 ± 0.4
Visible light during sleep (log lux)	0.04 ± 0.02	0.03 ± 0.02
Blue light during sleep (log lux)	0.02 ± 0.02	0.01 ± 0.01
Visible light 2h before SO (log lux)	0.53 ± 0.14	0.31 ± 0.08
Blue light 2h before SO (log lux)	0.17 ± 0.05	0.06 ± 0.02
Visible light 2h after waking (log lux)	1.19 ± 0.18	0.96 ± 0.14
Blue light 2h after waking (log lux)	0.66 ± 0.11	0.59 ± 0.10
Napping time (min)	31.2 ± 22.5	11.4 ± 7.8

Data expressed as mean ± SEM. AT, awakening time (last time period marked as sleep before the get up time); SE, percentage of time asleep with regard to the time in bed; SL, total time in minutes between bed time and SO; SO, sleep onset (first time period marked as sleep after bed time); TIB, period of time between bed time and get up time; TST, time marked as sleep during the sleep interval; TTM, total minutes in the sleep interval during which movement has been detected; WASO, wake after sleep onset time (total minutes marked as awake after SO); WT, wrist skin temperature. A student’s t-test was used to compare CLP and NO CLP means for each parameter. P-values were corrected for multiple testing with the Benjamini-Hochberg method. No significant differences were found. A two-way ANOVA was conducted to analyse possible interactions between group (CLP or NO CLP) and gender. No significant differences were found.

### 3.3 Blind participants versus controls

**Figure 2:**
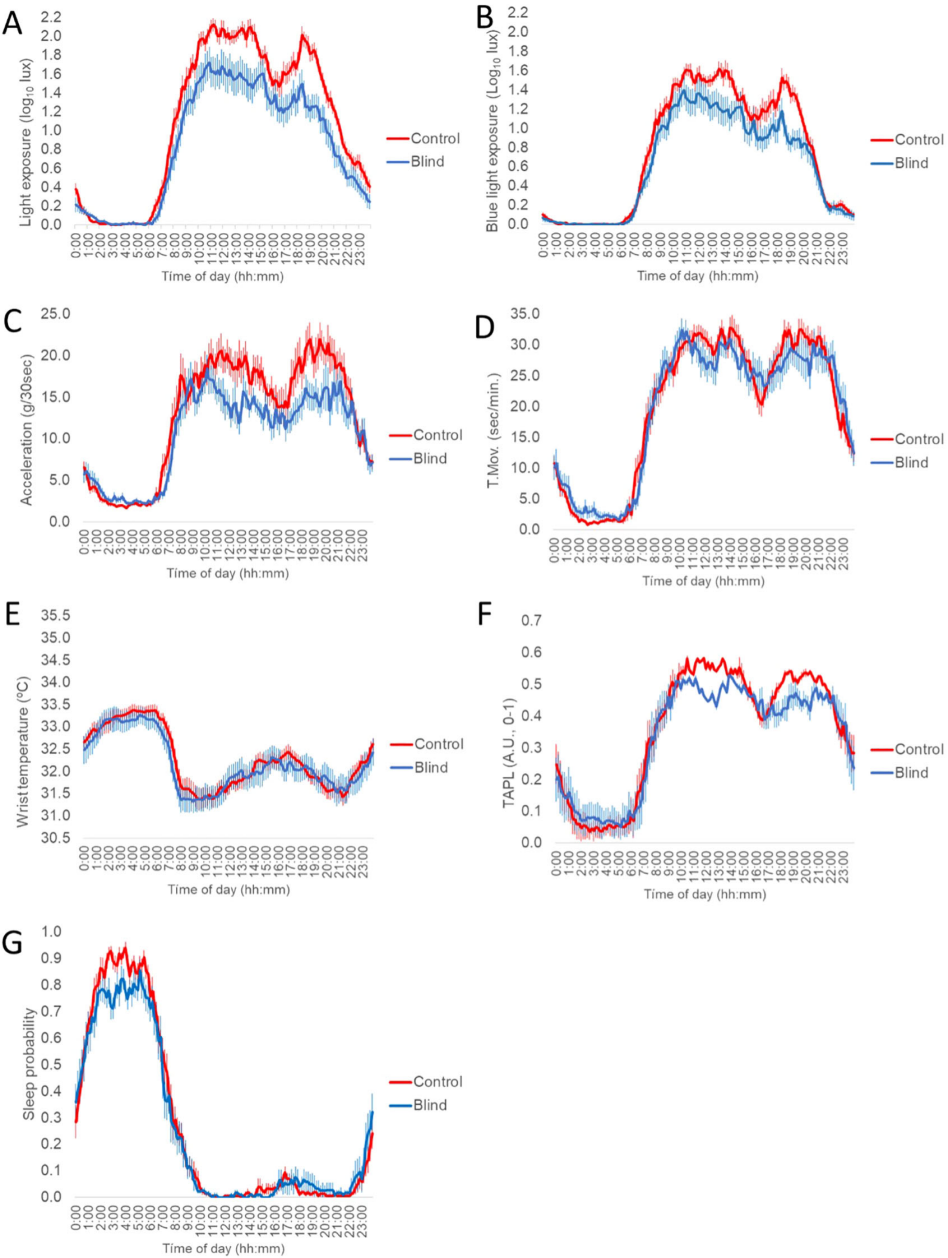
Mean-waveforms for total blind (blue line, n=18) and controls subjects (red line, n=26) for: **(A)** visible light exposure; **(B)** blue light exposure; **(C)** acceleration of movement; **(D)** time in movement; **(E)** distal skin temperature; **(F)** the integrated variable TAPL and **(G)** sleep probability. All variables are expressed as the mean ± SEM (vertical bars).

Daily mean patterns of all recorded variables from blind subjects under free living conditions and their controls are shown in **Figure 2**, while their rhythmic characteristics are detailed in **Table 2**. As expected, the rhythms of environmental light exposure (LE, visible light; BLE, blue light) showed high exposure levels during the day and low exposure levels during the night (**Figures 2A, B**). Blind participants showed significantly lower LE during the day, particularly indoors light (M10 for total LE, *p=0.038;* Indoors*, p=0.038;* Outdoors*, 0.055*) (**Supplementary Table 2A**) and NRA (*p=0.038*) than controls (**Table 2**). Besides, blind subjects had lower LE levels in the 2h after waking (*p<0.001*) than controls (**Table 3**). During the night, no statistical differences in darkness length (442.0 ± 14.7 min for blind participants and 440.6 ± 8.4 for controls) and midpoint of the lowest light exposure (LE L5T, **Table 2**) between blind participants and controls were found.

On the other hand, acceleration (A) and time in movement (TM) rhythms exhibited low values at night and high values during daytime (**Figures 2C, D**, respectively). Blind participants showed significantly lower A values during the day (M10, *p=0.014*) (**Table 2**) and therefore, a significantly lower A/T index than controls (*p=0.024*) (**Table 3**). Besides, blind subjects showed lower A NRA (*p=0.010*) and A CHS (*p=0.018*) than controls. When participants’ physical activity was categorized according to intensity, we found that blind participants spent more time in sedentary activities (*p=0.018*) and less time in moderate activities (*p<0.010*) during daytime than controls (**Supplementary Table 3A**).

The distal skin temperature (WT) pattern of blind participants and controls (**Figure 2E**) showed common rhythmic characteristics, with high and relatively stable values during sleep time and low and highly variable values during the active phase. When compared, no differences in circadian parameters between the blind and control populations were found.

The integrated variable TAPL (general activation) showed low and stable values at night, and high and variable values during the daytime. (**Figure 2F**). TAPL rhythm from the blind participants had significantly lower values of NRA (*p=0.047*) and, consequently, lower CHS (*p=0.047*) than that from controls (**Table 2**). Also, a tendency to higher values during the night (L5, *p=0.054*) and lower regularity (IS, *p=0.054*) was appreciated.

Finally, the sleep probability rhythm showed high values during the night and low values during daytime, as expected, with an afternoon peak associated to postprandial somnolence (**Figure 2G**). Although night sleep length (360.0 ± 14.0 min for blind volunteers and 377.7 ± 7.8 min for controls) and midpoint (Sleep M5T, **Table 2**) were not significantly different between blind volunteers and controls, blind subjects had a lower sleep probability during the night (M5, *p=0 018*) and lower values of NRA (*p=0.030*) than controls (**Table 2**). Besides, blind participants showed a significantly lower sleep efficiency (higher awake time during the sleep period, *p=0.002*). Lastly, the regularity of sleep rhythm tended to be lower in blind participants (IS, *p=0.068).*

### 3.4 CLP versus NO CLP

**Figure 3:**
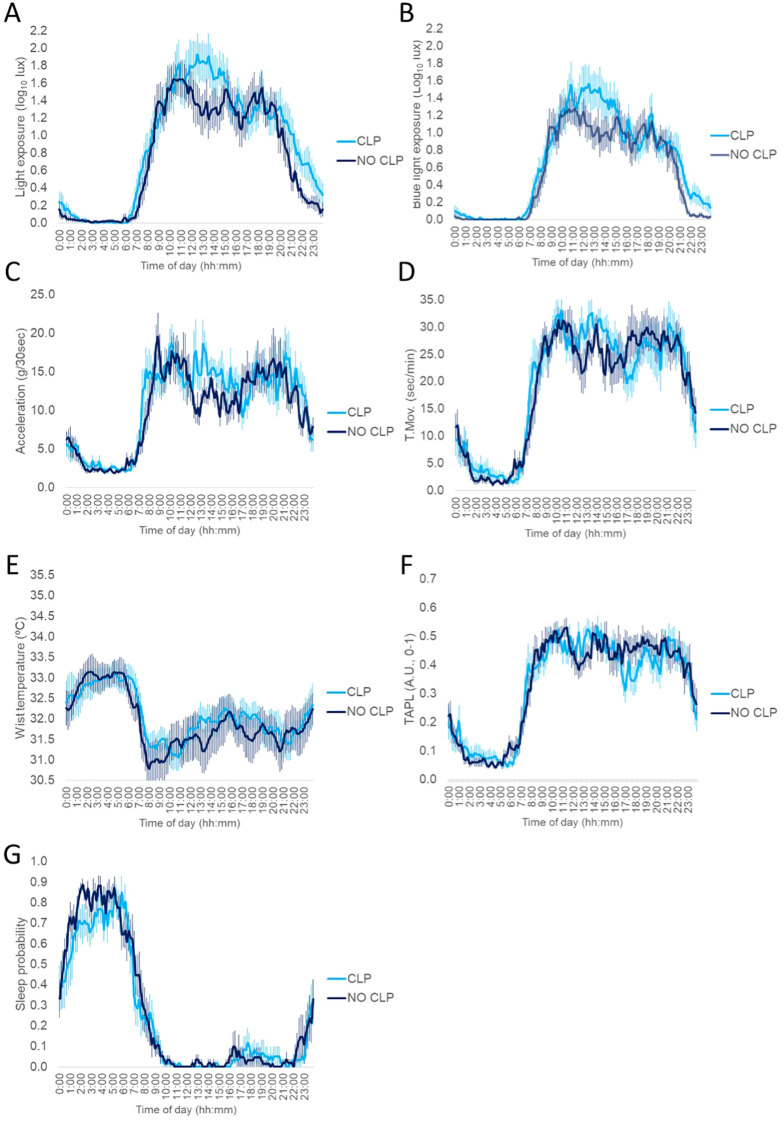
Mean-waveforms for blind participants with conscious light perception (CLP) (light blue line, n=9) and blind subjects with no conscious light perception (NO CLP) (dark blue, n=9) for: **(A)** visible light exposure; **(B)** blue light exposure; **(C)** acceleration of movement; **(D)** time in movement; **(E)** distal skin temperature; **(F)** the integrated variable TAPL and **(G)** sleep. All variables are expressed as the mean ± SEM.

Daily mean patterns of all recorded circadian rhythms from blind participants with CLP and with NO CLP were very similar (**Figure 3**). When we compared non-parametric indexes from the two groups, we found that WT rhythm was delayed during the night in NO CLP compared to CLP (M5T, 3.03 ± 0.54 vs 7.47 ± 1.90) being coherent with a worse environmental synchronization in this group (ES 0.87 ± 0.03 vs 0.61 ± 0.13) but none of these differences reached significance after correction (**Table 4**, **Supplementary Table 1**). On the other hand, no differences for LE, BLE, A, TM and sleep rhythms were found for any circadian parameter.

Time spent in in- and outdoors light (**Supplementary Table 2B**) and in physical activities of different intensities (**Supplementary Table 3B**) was not statistically different between CLP and NO CLP either.

Regarding sleep parameters, no significant differences were found after multiple test correction. However, on average, blind participants with CLP seemed to be more exposed to blue light during the 2h before sleep onset (0.17 ± 0.05 vs 0.06 ± 0.02). CLP participants also tended to have an increased awakening rate during the night (3.3 ± 0.3 vs 2.3 ± 0.2) and a higher inner synchronization (IntS,1.08 ± 0.04 vs 0.69 ± 0.16) than those with NO CLP (**Table 5**).

**Figure 4:**
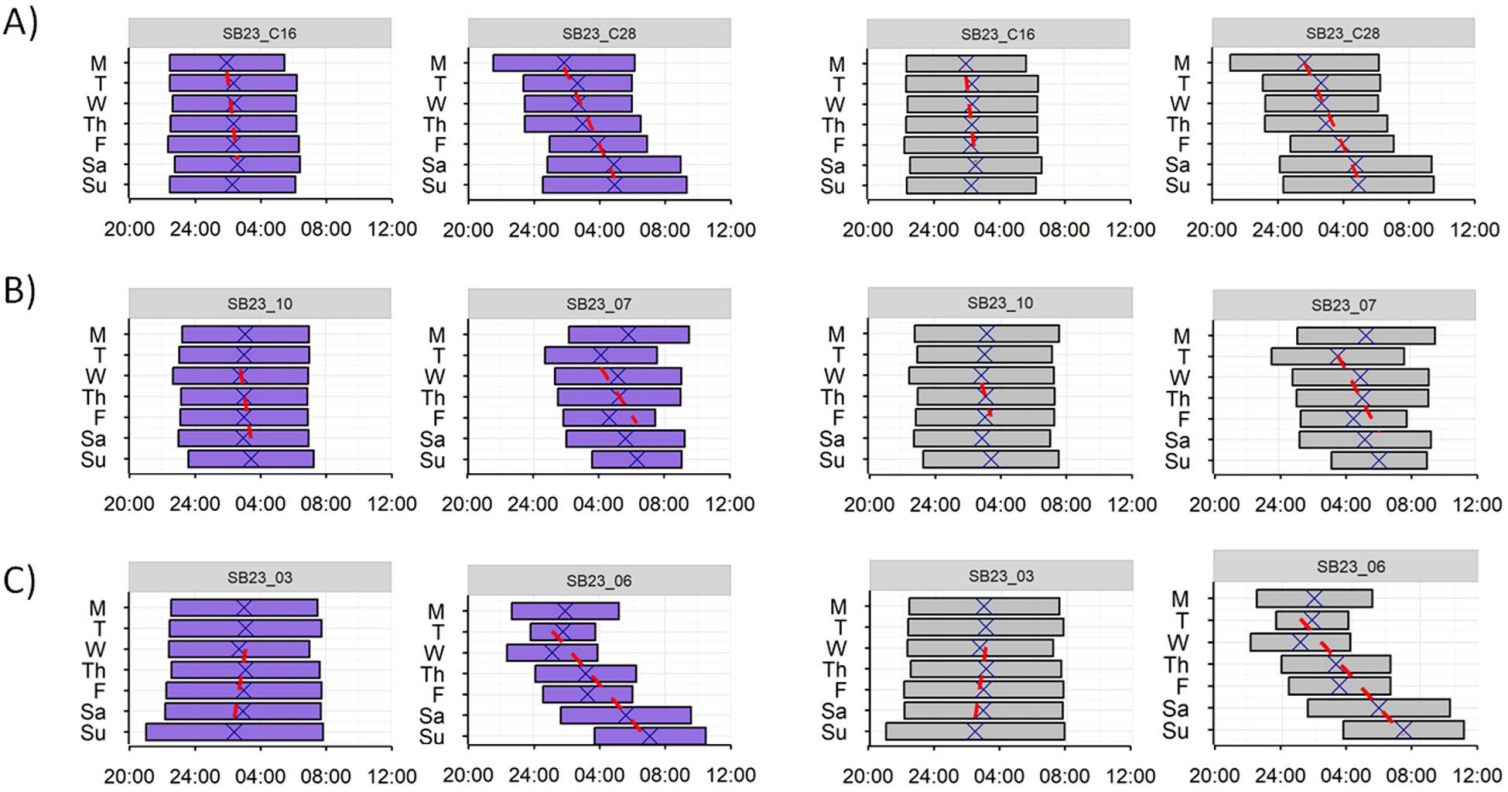
Individual length (bars) and midpoint (crosses) of night sleep (purple) and darkness (grey) during 7 consecutive days (M: Monday; T: Tuesday; W: Wednesday; Th: Thursday; F: Friday; Sa: Saturday; Su: Sunday) of two control participants **(A)**, two blind participants with conscious light perception **(B)** and two blind participants with no conscious light perception **(C)**. Red lines indicate midpoints trend across the seven days.

Length and midpoint of night sleep and darkness were represented for every volunteer during the 7 days of the recordings (**Figure 4**). For every group analysed (control, CLP and NO CLP), we found participants with entrained sleep rhythms (like SB23-C16) and subjects whose sleep patterns seemed to free run along the days (see for example SB23-C28). Besides, in most of the recordings, darkness exposure patterns were almost identical to those of sleep.

### 3.5 Night fasting

**Figure 5:**
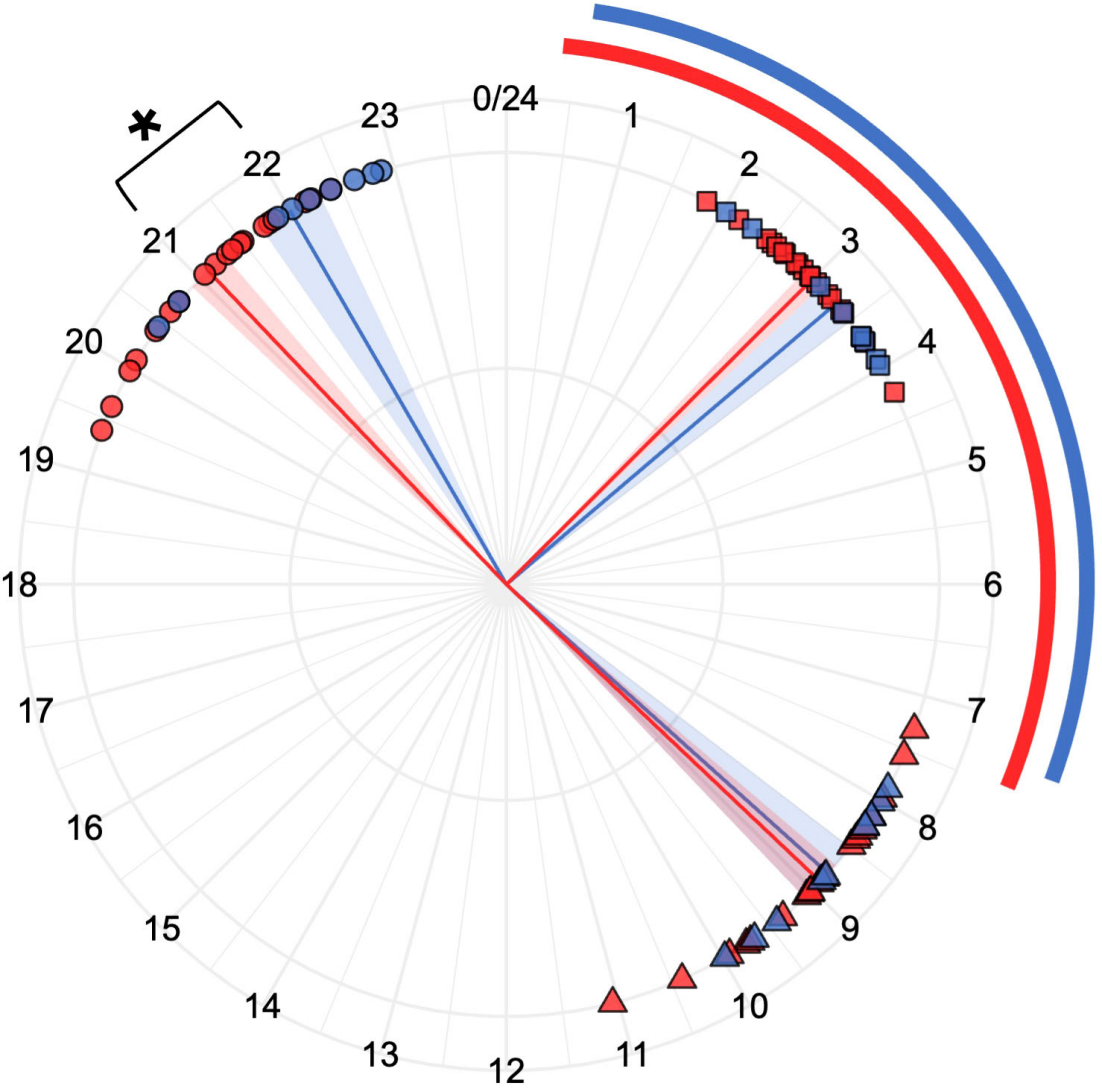
Night fasting of Blind (n=9, colour blue) and Control (n=20, colour red) participants. Individual values of last meal (circles), mid-fasting (squares) and first meal (triangles) are represented. Lines and shades correspond to the mean and the standard error of mean, respectively. Around graph, their corresponding average night sleep periods are shown with bars. A Student’s t-test was used to compare Blind and Control participants means for each parameter. * indicates p-adjusted< 0.05.

Night fasting length was significantly shorter in blind participants than in controls (646.0 ± 21.3 vs 715.1 ± 20.6 minutes; *p= 0.037*) (**Figure 5**). The blind subjects’ last meal of the day happened later (22.0 ± 0.3 vs 21.1 ± 0.2; *p: 0.034*) and was more regular than that of controls, showing lower values of Composite Phase Deviation (1.0 ± 0.2 vs 1.8 ± 0.3 hours; *p: 0.034*), while there were no statistical differences in the first meal of the days’ time and regularity. Considering that sleep length and midpoints were not statistically different between both groups, the time between the last meal of the day and either the sleep onset (2.5 ± 0.3 vs 3.3 ± 0.2 hours; *p: 0.058*) and the sleep midpoint (5.9 ± 0.3 vs 6.9 ± 0.2 hours; *p: 0.034*) was shorter in the blind participants than in controls.

## 4 Discussion

In general, blind participants showed poorer circadian function than controls, with most of the analysed rhythms presenting lower amplitude. Surprisingly, blind volunteers did not show significant differences in the circadian rhythms’ phase or in their environmental synchronization (ES). When the daily sleep onset, midpoint and offset of each individual were represented, we found that the two most evident cases of free-running sleep among the blind volunteers corresponded to individuals with bilateral enucleation. Among the blind individuals who retained eyes, we found some less patent cases that could match with a free running sleep rhythm in both, blind participants who declared having some sort of light perception (CLP) and those who lacked conscious light perception (NO CLP).

This study has shown us the enormous variability in the aetiology of blindness. We have found up to eight different causes of blindness in a sample of eighteen participants and, except for the two individuals who had lost both eyes, we cannot be certain up to what extent ipRGCs or the retino-hypothalamic tract (RHT) are impacted in each condition since it is not usually assessed in the clinical practice, and thus it is not generally consigned in the clinical history. Furthermore, it must be considered that even individuals with the same diagnosed ocular disease can have different visual (i.e. keeping or lacking CLP) and circadian phenotype, as already described (Hull et al., 2018). Therefore, we will need for future work to determine exactly how melanopsin and ipRGC are impacted by visual diseases and the development of a robust biomarker for personalized evaluation of the circadian photoreception function is an obvious starting point (Allen, 2019).

To make the picture even more complicated, we also found some cases of free-running sleep in the control population, which remarks the fact that circadian photoreception does not guarantee entrainment since daily habits, not only light exposure but also sleep patterns, physical exercise or feeding times among others, are important contributors (Lazreg et al., 2011; St. Hilaire et al., 2007). If measured outside lab conditions, confounding factors can disguise read-outs of circadian rhythms, for example, sleep/wake cycles. Depending on the phase of the individual’s circadian clock, participants with non-24-hour sleep–wake disorders may show symptoms of delayed or advanced phases, or no circadian abnormalities whatsoever. Therefore, the diagnosis of circadian disorders in the blind participants in real-life was an additional challenge of this study.

Hartley et al. (2018) have stated that the key tool to investigate circadian rhythm disorders is an objective record of sleep and wake times over an extended period (weeks or even months when necessary) and actigraphy is recommended in patients for whom there is a suspicion of a circadian rhythm disturbance. This wearable technology opens the possibility to study, on a long-term basis, a wide range of people in real-time conditions and provides, at the same time, information on a single individual that can then be used to guide health-related decisions. But there are other objective approaches that improve the performance of actigraphy alone (Almaida-Pagan et al., 2022). Ambulatory circadian monitoring (ACM), thanks to sensors included in the wristwatch-like Kronowise^®^, combines measurements of a) endogenous variables, such as skin temperature (Sarabia et al., 2008); b) *zeitnehmers* (Roenneberg et al., 2003) (German word meaning timekeeper), such as motor activity and body position more dependent on willingness (Skeldon et al., 2017); and c) exogenous synchronisers or *zeitgebers*, such as light exposure. In fact, KW includes three light sensors to record visible, infrared and blue light. The sensor for blue light is equipped with a filter that only let circadian light through (Arguelles-Prieto et al., 2019), which is the one primarily detected by the ipRGC. The ratio between infrared/visible light makes it possible to determine the source of light (i.e. natural, fluorescent, infrared, incandescent, or LED light), providing information about life-style and the bidirectional crosstalk between internal time and external synchronisers (Arguelles-Prieto et al., 2019; Madrid-Navarro et al., 2015; Martinez-Nicolas et al., 2011), with especial focus on light exposure, which is paramount to determine a subject suffering from chronodisruption (Erren and Reiter, 2013). It is true that light is measured at wrist level, but according to literature it can provide accurate light measurements relative to locating a calibrated photosensor at the plane of the cornea (Figueiro et al., 2013). Besides, ACM has been validated for sleep/wake detection (it shows higher sensitivity and specificity than actigraphy alone) by comparison with sleep diaries (Ortiz-Tudela et al., 2010) and polysomnography (PSG) (Ortiz-Tudela et al., 2014a) and it can be used instead of DLMO (dim light melatonin onset) to predict the internal phase (Bonmati-Carrion et al., 2014). ACM has proven its usefulness in very different populations, such as shift workers (Gomez-Garcia et al., 2016; Moreno-Casbas et al., 2014), babies (Batinga et al., 2015; Zornoza-Moreno et al., 2011), hypertensive and patients with metabolic syndrome (Blazquez et al., 2012; Corbalán-Tutau et al., 2011), ageing (Batinga et al., 2015; Martinez-Nicolas et al., 2018), mild cognitive impairment (Ortiz-Tudela et al., 2014b), sleep-disordered breathing (Martinez-Nicolas et al., 2017), Parkinson´s disease (Madrid-Navarro et al., 2018) and cancer (Almaida-Pagan et al., 2022), among others.

As determined by ACM, blindness is linked to disruptions in behaviour, as we can see in light exposure, with blind participants showing significantly lower visible light exposure during the day and thus, reduced amplitude of daily light-dark contrast, and physical activity, with blind subjects showing lower activity values during the day (mainly referred to acceleration or intensity), which correlates with poorer sleep efficiency. These results would agree with previous data showing poorer sleep quality in the blind population (Lazreg et al., 2011; Medeiros da Silva et al., 2016) and our methodology would point to light exposure and physical activity as behavioural approaches to improve circadian and sleep status of the blind population in future interventions.

For the first time, we analysed blind volunteers’ feeding times over a week prospectively and found that their last meal of the day happened later than in controls and blind participants’ night fasting was shorter. Apart from inner, environmental and social times, feeding schedules play an important role in regulating peripheral clocks and metabolism (Lazreg et al., 2011; Lockley et al., 2007; Sinturel et al., 2021; St. Hilaire et al., 2007; Wehrens et al., 2017) and thus, more attention to this aspect will be necessary in future research.

Interestingly, women showed higher wrist temperature during the night than men, this being particularly marked in blind subjects. This would agree with data from a previous study, in which adult women showed a more robust WT pattern (lower fragmentation, and higher night-time temperature, amplitude, circadian function index, and first harmonic relative power) than men, although these differences were lost with ageing, a period of life that was consistently associated with a phase advance of the rhythm (Batinga et al., 2015).

When subjects with CLP and NO CLP were compared, we found no significant differences in circadian parameters for any analysed rhythm. Only an interesting tendency was appreciated for distal skin temperature from blind participants, with NO CLP having a delayed phase of nocturnal WT values regarding that of blind subjects with CLP. This result might indicate that people lacking CLP are prone to have worse environmental synchronization of the most endogenous circadian rhythm analysed. These data, if confirmed with future studies, would point to possible alterations of the temperature rhythm in blind subjects with NO CLP, which is directly regulated by the central clock, and circadian alterations that were not clearly reflected on sleep.

Summarizing, this study shows promising data pointing to poorer circadian health in the blind participants, which could be directly related to the impact of disease over circadian photoreception and their self-selected light exposure but also to disruption of daily habits that could also input the circadian system. ACM allows for a multidimensional analysis of circadian function and sleep in real life, considering light exposure and behaviour. For future studies, we will need to increase the number of participants to address important questions such as the effect of age of the onset of blindness or the relationship between specific ocular diseases and varying degrees of sleep/circadian disturbances. It will be also necessary to objectively measure ipRGC and RHT integrity, e.g. measuring the regulation of pupil size (Bonmati-Carrion et al., 2016), as an initial screening in blind individuals. Better diagnoses should in turn lead to a clearer understanding of how blindness impacts circadian photoentrainment. As a result, we will be able to recognise how the most promising therapeutic approaches can be implemented.

## Data Availability

The raw data supporting the conclusions of this article will be made available by the authors, without undue reservation.

## References

[B1] Adeoti A. (2010). Disorders of the sleep-wake cycle in blindness. West. Afr. J. Med. 29, 163–168. doi: 10.1111/j.1365-2214.2010.01112.x. PMID: 20665459

[B2] Allen A. E. (2019). Circadian rhythms in the blind. Curr. Opin. Behav. Sci. 30, 73–79. doi: 10.1016/J.COBEHA.2019.06.003. PMID: 41836151

[B3] Almaida-Pagan P. F. Torrente M. Campos M. Provencio M. Madrid J. A. Franco F. . (2022). Chronodisruption and ambulatory circadian monitoring in cancer patients: Beyond the body clock. Curr. Oncol. Rep. 24, 135–149. doi: 10.1007/s11912-021-01158-z. PMID: 35061192 PMC8857092

[B4] American Academy of Sleep Medicine (2014). International classification of sleep disorders (3rd ed.). Darien, IL: American Academy of Sleep Medicine.

[B5] Arguelles-Prieto R. Bonmati-Carrion M. A. Rol M. A. Madrid J. A. (2019). Determining light intensity, timing and type of visible and circadian light from an ambulatory circadian monitoring device. Front. Physiol. 10. doi: 10.3389/fphys.2019.00822. PMID: 31297069 PMC6607467

[B6] Batinga H. Martinez-Nicolas A. Zornoza-Moreno M. Sánchez-Solis M. Larqué E. Mondéjar M. T. . (2015). Ontogeny and aging of the distal skin temperature rhythm in humans. Age 37, 29. doi: 10.1007/s11357-015-9768-y. PMID: 25813804 PMC4375132

[B7] Berson D. M. Dunn F. A. Takao M. (2002). Phototransduction by retinal ganglion cells that set the circadian clock. Sci. (80-.) 295, 1070–1073. doi: 10.1126/science.1067262. PMID: 11834835

[B8] Blazquez A. Martinez-Nicolas A. Salazar F. J. Rol M. A. Madrid J. A. (2012). Wrist skin temperature, motor activity, and body position as determinants of the circadian pattern of blood pressure. Chronobiol. Int. 29, 747–756. doi: 10.3109/07420528.2012.679328. PMID: 22734575

[B9] Bonmati-Carrion M. A. Hild K. Isherwood C. Sweeney S. J. Revell V. L. Skene D. J. . (2016). Relationship between human pupillary light reflex and circadian system status. PloS One 11, 1–21. doi: 10.1371/journal.pone.0162476. PMID: 27636197 PMC5026360

[B10] Bonmati-Carrion M. A. Middleton B. Revell V. Skene D. J. Rol M. A. Madrid J. A. (2014). Circadian phase asessment by ambulatory monitoring in humans: Correlation with dim light melatonin onset. Chronobiol. Int. 31, 37–51. doi: 10.3109/07420528.2013.820740. PMID: 24164100

[B11] Canteras N. S. Ribeiro-Barbosa É.R. Goto M. Cipolla-Neto J. Swanson L. W. (2011). The retinohypothalamic tract: Comparison of axonal projection patterns from four major targets. Brain Res. Rev. 65 (2), 150–183. doi: 10.1016/j.brainresrev.2010.09.006. PMID: 20863850

[B12] Corbalán-Tutau M. D. Madrid J. A. Ordovás J. M. Smith C. E. Nicolás F. Garaulet M. (2011). Differences in daily rhythms of wrist temperature between obese and normal-weight women: Associations with metabolic syndrome features. Chronobiol. Int. 28, 425–433. doi: 10.3109/07420528.2011.574766. PMID: 21721858 PMC4372336

[B13] Czeisler C. A. (2013). Perspective: Casting light on sleep deficiency. Nature 497, S13. doi: 10.1038/497S13a. PMID: 23698501

[B14] Das A. Sasmal N. K. Deb R. K. Bera N. K. Sanyal D. Chatterjee S. S. . (2006). A study of sleeping disorders in blind patients. J. Indiano Med. Assoc. 104, 619–626. 17444060

[B15] Davies W. L. Foster R. G. Hankins M. W. (2012). Focus on molecules: melanopsin. Exp. Eye Res. 97, 161–162. doi: 10.1016/j.exer.2010.07.020. PMID: 20696158

[B16] Erren T. C. Morfeld P. Stork J. Knauth P. Mülmann M. V. Breitstadt R. . (2009). Shift work, chronodisruption and cancer? - The IARC 2007 challenge for research and prevention and 10 theses from the Cologne Colloquium 2008. Scand. J. Work. Environ. Heal. 35, 74–79. doi: 10.5271/sjweh.1303. PMID: 19277435

[B17] Erren T. C. Reiter R. J. (2013). Revisiting chronodisruption: When the physiological nexus between internal and external times splits in humans. Naturwissenschaften 100, 291–298. doi: 10.1007/s00114-013-1026-5. PMID: 23494200

[B18] Erren T. C. Reiter R. J. Piekarski C. (2003). Light, timing of biological rhythms, and chronodisruption in man. Naturwissenschaften. 90, 485–494. doi: 10.1007/s00114-003-0468-6. PMID: 14610644

[B19] Figueiro M. G. Hamner R. Bierman A. Rea M. S. (2013). Comparisons of three practical field devices used to measure personal light exposures and activity levels. Light. Res. Technol. 45, 421–434. doi: 10.1177/1477153512450453. PMID: 24443644 PMC3892948

[B20] Fischer D. Vetter C. Roenneberg T. (2016). A novel method to visualise and quantify circadian misalignment. Sci. Rep. 6, 1–10. doi: 10.1038/srep38601. PMID: 27929109 PMC5144069

[B21] Garaulet M. Madrid J. A. (2010). Chronobiological aspects of nutrition, metabolic syndrome and obesity. Adv. Drug Deliv. Rev. 62 (9–10), 967–978. doi: 10.1016/j.addr.2010.05.005. PMID: 20580916

[B22] Gomez-Garcia T. Ruzafa-Martinez M. Fuentelsaz-Gallego C. Madrid J. A. Rol M. A. Martinez-Madrid M. J. . (2016). Nurses’ sleep quality, work environment and quality of care in the Spanish National Health System: Observational study among different shifts. BMJ Open 6, 1–11. doi: 10.1136/bmjopen-2016-012073. PMID: 27496241 PMC4985858

[B23] Gómez-Ulla de Irazazábal F. Odategui-Parra S. (2012). Informe sobre la ceguera en España.

[B24] Grone B. P. Chang D. Bourgin P. Cao V. Fernald R. D. Heller H. C. . (2011). Acute light exposure suppresses circadian rhythms in clock gene expression. J. Biol. Rhythms 26, 78–81. doi: 10.1177/0748730410388404. PMID: 21252368

[B25] Hankins M. W. Peirson S. N. Foster R. G. (2008). Melanopsin: an exciting photopigment. Trends Neurosci. 31 (1), 27–36. doi: 10.1016/j.tins.2007.11.002. PMID: 18054803

[B26] Hartley S. Dauvilliers Y. Quera-Salva M. A. (2018). Circadian rhythm disturbances in the blind. Curr. Neurol. Neurosci. Rep. 18, 65. doi: 10.1007/s11910-018-0876-9. PMID: 30083814

[B27] Haynes W. (2013). Benjamini–hochberg method. Encycl. Syst. Biol., 78–78. doi: 10.1007/978-1-4419-9863-7_1215. PMID: 41739277

[B28] Hull J. T. Czeisler C. A. Lockley S. W. (2018). Suppression of melatonin secretion in totally visually blind people by ocular exposure to white light: Clinical characteristics. Ophthalmology 125, 1160–1171. doi: 10.1016/j.ophtha.2018.01.036. PMID: 29625838

[B29] International Agency for Research on Cancer (WHO) (2020). Night shift work, IARC Monographs on the identification of carcinogenic hazards to humans (Lyon, France: International Agency for Research on Cancer).

[B30] Kantermann T. (2013). Circadian biology: Sleep-styles shaped by light-styles. Curr. Biol. 23, R689–R690. doi: 10.1016/j.cub.2013.06.065. PMID: 23968925

[B31] Karatsoreos I. N. Bhagat S. Bloss E. B. Morrison J. H. McEwen B. S. (2011). Disruption of circadian clocks has ramifications for metabolism, brain, and behavior. Proc. Natl. Acad. Sci. U.S.A. 108, 1657–1662. doi: 10.1073/pnas.1018375108. PMID: 21220317 PMC3029753

[B32] Kawasaki A. Kardon R. (2007). Intrinsically photosensitive retinal ganglion cells. J. Neuro Ophthalmol. 27 (3), 195–204. doi: 10.1097/WNO.0b013e31814b1df9. PMID: 17895821

[B33] Lazreg T. B. Laatiri I. Dogui M. (2011). Circadian activity-rest and sleep-wake rhythms in blind adolescents and adults. Biol. Rhythm. Res. 42, 219–229. doi: 10.1080/09291016.2010.500869. PMID: 41799851

[B34] Leger D. Guilleminault C. DeFrance R. Domont A. Paillard M. (1996). Blindness and sleep patterns. Lancet 348, 830–831. doi: 10.1016/s0140-6736(05)65256-7. PMID: 8814013

[B35] Lockley S. W. Arendt J. Skene D. J. (2007). Visual impairment and circadian rhythm disorders. Dialogues Clin. Neurosci. 9, 301–314. doi: 10.31887/dcns.2007.9.3/slockley. PMID: 17969867 PMC3202494

[B36] Madrid-Navarro C. J. Cuesta F. J. P. Escamilla-Sevilla F. Campos M. Abellán F. R. Rol M. A. . (2019). Validation of a device for the ambulatory monitoring of sleep patterns: A pilot study on Parkinson’s disease. Front. Neurol. 10. doi: 10.3389/fneur.2019.00356. PMID: 31031690 PMC6470193

[B37] Madrid-Navarro C. J. Escamilla-Sevilla F. Mínguez-Castellanos A. Campos M. Ruiz-Abellán F. Madrid J. A. . (2018). Multidimensional circadian monitoring by wearable biosensors in Parkinson’s disease. Front. Neurol. 9. doi: 10.3389/fneur.2018.00157. PMID: 29632508 PMC5879441

[B38] Madrid-Navarro C. J. Sanchez-Galvez R. Martinez-Nicolas A. Marina R. García J. A. Madrid J. A. . (2015). Disruption of circadian rhythms and delirium, sleep impairment and sepsis in critically ill patients. Potential therapeutic implications for increased light-dark contrast and melatonin therapy in an ICU environment. Curr. Pharm. Des. 21, 3453–3468. doi: 10.2174/1381612821666150706105602. PMID: 26144941

[B39] Martinez-Nicolas A. Almaida-Pagan P. F. Martinez-Madrid M. J. Arguelles-Prieto R. Ortega-Sabater C. Fernandez-Ortiz M. . (2018). “ Ageing of the circadian system. From monitoring to chronoenhancement,” in Approaches to Aging Control. Eds. Ayala A. Serres J. (Seville, Spain: SEMAL), 62–72.

[B40] Martinez-Nicolas A. Guaita M. Santamaría J. Montserrat J. M. Rol M. Á. Madrid J. A. (2017). Circadian impairment of distal skin temperature rhythm in patients with sleep-disordered breathing: The effect of CPAP. Sleep 40, zsx067. doi: 10.1093/sleep/zsx067. PMID: 28444396

[B41] Martinez-Nicolas A. Martinez-Madrid M. J. Almaida-Pagan P. F. Bonmati-Carrion M.-A. Madrid J. A. Rol M. A. (2019). Assessing chronotypes by ambulatory circadian monitoring. Front. Physiol. 10. doi: 10.3389/fphys.2019.01396. PMID: 31824327 PMC6879660

[B42] Martinez-Nicolas A. Ortiz-Tudela E. Madrid J. A. Rol M. A. (2011). Crosstalk between environmental light and internal time in humans. Chronobiol. Int. 28, 617–629. doi: 10.3109/07420528.2011.593278. PMID: 21793693

[B43] Maywood E. S. O’Neill J. S. Reddy A. B. Chesham J. E. Prosser H. M. Kyriacou C. P. . (2007). “ Genetic and molecular analysis of the central and peripheral circadian clockwork of mice,” in Cold Spring Harbor Symposia on Quantitative Biology. New York, USA: Cold Spring Harbor Laboratory Press. 85–94. doi: 10.1101/sqb.2007.72.005, PMID: 18419265

[B44] Mazzoccoli G. Giuliani F. Sothern R. B. (2011). A method to evaluate dynamics and periodicity of hormone secretion. J. Biol. Regul. Homeost. Agents 25, 231–238. 21880212

[B45] Medeiros da Silva K. K. Figueiredo De Martino M. M. Pereira Cruz G. K. Ferreira Junior M. A. Brandão de Carvalho Lira A. L. Fortes Vitor A. . (2016). Sleep disorders related to circadian rhythm in people with visual impairment: Integrative review. Int. Arch. Med. 9. doi: 10.3823/2238, PMID: 42030518

[B46] Mehta R. Zhu R. J. (2009). Blue or red? Exploring the effect of color on cognitive task performances. Science 323, 1226–1229. doi: 10.1126/science.1169144, PMID: 19197022

[B47] Moore R. Y. Speh J. C. (2004). Serotonin innervation of the primate suprachiasmatic nucleus. Brain Res. 1010, 169–173. doi: 10.1016/j.brainres.2004.02.024. PMID: 15126131

[B48] Moreno-Casbas M. T. Ruzafa-Martinez M. Rol M. A. Madrid J. A. Pinto A. S. Gonzalez-Maria E. . (2014). Sleepiness in Spanish nursing staff - Influence of chronotype and care unit in circadian rhythm impairment: Research protocol. J. Adv. Nurs. 70, 211–219. doi: 10.1111/jan.12200. PMID: 23834526

[B49] Morin L. P. Shivers K. Y. Blanchard J. H. Muscat L. (2006). Complex organization of mouse and rat suprachiasmatic nucleus. Neuroscience 137, 1285–1297. doi: 10.1016/j.neuroscience.2005.10.030. PMID: 16338081

[B50] Ortiz-Tudela E. Martinez-Nicolas A. Albares J. Segarra F. Campos M. Estivill E. . (2014a). Ambulatory circadian monitoring (ACM) based on thermometry, motor activity and body position (TAP): A comparison with polysomnography. Physiol. Behav. 126, 30–38. doi: 10.1016/j.physbeh.2013.12.009. PMID: 24398067

[B51] Ortiz-Tudela E. Martinez-Nicolas A. Campos M. Rol M. Á. Madrid J. A. (2010). A new integrated variable based on thermometry, actimetry and body position (TAP) to evaluate circadian system status in humans. PloS Comput. Biol. 6, e1000996. doi: 10.1371/journal.pcbi.1000996. PMID: 21085644 PMC2978699

[B52] Ortiz-Tudela E. Martinez-Nicolas A. Díaz-Mardomingo C. García-Herranz S. Pereda-Pérez I. Valencia A. . (2014b). The characterization of biological rhythms in mild cognitive impairment. BioMed. Res. Int. 2014, 1–7. doi: 10.1155/2014/524971. PMID: 25157363 PMC4124835

[B53] Raymann R. J. E. M. Swaab D. F. Van Someren E. J. W. (2005). Cutaneous warming promotes sleep onset. Am. J. Physiol. Regul. Integr. Comp. Physiol. 288, 1589–1597. doi: 10.1152/ajpregu.00492.2004. PMID: 15677527

[B54] Refinetti R. Cornélissen G. Halberg F. (2007). Procedures for numerical analysis of circadian rhythms. Biol. Rhythm. Res. 38 (4), 275–325. doi: 10.1080/09291010600903692. PMID: 23710111 PMC3663600

[B55] Roenneberg T. Wirz-Justice A. Merrow M. (2003). Life between clocks: Daily temporal patterns of human chronotypes. J. Biol. Rhythms 18, 80–90. doi: 10.1177/0748730402239679. PMID: 12568247

[B56] Sarabia J. A. Rol M. A. Mendiola P. Madrid J. A. (2008). Circadian rhythm of wrist temperature in normal-living subjects. A candidate of new index of the circadian system. Physiol. Behav. 95, 570–580. doi: 10.1016/j.physbeh.2008.08.005. PMID: 18761026

[B57] Sinturel F. Gos P. Petrenko V. Hagedorn C. Kreppel F. Storch K. F. . (2021). Circadian hepatocyte clocks keep synchrony in the absence of a master pacemaker in the suprachiasmatic nucleus or other extrahepatic clocks. Genes Dev. 35, 329–334. doi: 10.1101/gad.346460.120. PMID: 33602874 PMC7919413

[B58] Skeldon A. C. Phillips A. J. K. Dijk D. J. (2017). The effects of self-selected light-dark cycles and social constraints on human sleep and circadian timing: A modeling approach. Sci. Rep. 7, 1–14. doi: 10.1038/srep45158. PMID: 28345624 PMC5366875

[B59] St. Hilaire M. A. Klerman E. B. Khalsa S. B. S. Wright K. P. Czeisler C. A. Kronauer R. E. (2007). Addition of a non-photic component to a light-based mathematical model of the human circadian pacemaker. J. Theor. Biol. 247, 583–599. doi: 10.1016/j.jtbi.2007.04.001. PMID: 17531270 PMC3123888

[B60] Straif K. Baan R. Grosse Y. Secretan B. El Ghissassi F. Bouvard V. (2007). Carcinogenicity of shift-work, painting, and fire-fighting. Lancet Oncol. 8 (12), 1065–1066. doi: 10.1016/S1470-2045(07)70373-X, PMID: 19271347

[B61] Tabandeh H. Lockley S. W. Buttery R. Skene D. J. DeFrance R. Arendt J. . (1998). Disturbance of sleep in blindness. Am. J. Ophthalmol. 126, 707–712. doi: 10.1016/s0002-9394(98)00133-0. PMID: 9822235

[B62] Vandewalle G. Schmidt C. Albouy G. Sterpenich V. Darsaud A. Rauchs G. . (2007). Brain responses to violet, blue, and green monochromatic light exposures in humans: Prominent role of blue light and the brainstem. PloS One 2, 1–10. doi: 10.1371/journal.pone.0001247. PMID: 18043754 PMC2082413

[B63] Vandewalle G. van Ackeren M. J. Daneault V. Hull J. T. Albouy G. Lepore F. . (2018). Light modulates oscillatory alpha activity in the occipital cortex of totally visually blind individuals with intact non-image-forming photoreception. Sci. Rep. 8, 16968. doi: 10.1038/s41598-018-35400-9. PMID: 30446699 PMC6240048

[B64] Van Someren E. J. W. (2003). Thermosensitivity of the circadian timing system. Sleep Biol. Rhythms 1, 55–64. doi: 10.1046/j.1446-9235.2003.00002.x. PMID: 41717205

[B65] Verma A. K. Singh S. Rizvi S. I. (2023). Aging, circadian disruption and neurodegeneration: Interesting interplay. Exp. Gerontol. 172, 112076. doi: 10.1016/J.EXGER.2022.112076. PMID: 36574855

[B66] Vervloed M. P. J. Hoevenaars E. Maas A. (2003). Behavioral treatment of sleep problems in a child with a visual impairment. Journal of Visual Impairment & Blindness 97 (1), 28–37. doi: 10.1177/0145482X0309700104. PMID: 41836481

[B67] Vicente-Martinez J. Gonzalez-Romero P. Almaida-Pagan P. F. Martinez-Nicolas A. Madrid J. A. Rol M. A. (2023). “ Integral analysis of circadian rhythms,” in Statistical Methods at the Forefront of Biomedical Advances. Ed. Larriba I. (Cham, Switzerland: Springer Nature Switzerland AG), 185–236.

[B68] Warman G. R. Pawley M. D. Bolton C. Cheeseman J. F. Fernando A. T. Arendt J. . (2011). Circadian-related sleep disorders and sleep medication use in the New Zealand blind population: An observational prevalence survey. PloS One 6, 281–297. doi: 10.1371/journal.pone.0022073. PMID: 21789214 PMC3138759

[B69] Wehrens S. M. T. Christou S. Isherwood C. Middleton B. Gibbs M. A. Archer S. N. . (2017). Meal timing regulates the human circadian system. Curr. Biol. 27, 1768–1775.e3. doi: 10.1016/J.CUB.2017.04.059. PMID: 28578930 PMC5483233

[B70] Witting W. Kwa I. H. Eikelenboom P. Mirmiran M. Swaab D. F. (1990). Alterations in the circadian rest activity rhythm in aging and Alzheimers disease. Biol. Psychiatry 27, 563–572. doi: 10.1016/0006-3223(90)90523-5. PMID: 2322616

[B71] Zornoza-Moreno M. Fuentes-Hernández S. Sánchez-Solis M. Rol M. Á. Larqué E. Madrid J. A. (2011). Assessment of circadian rhythms of both skin temperature and motor activity in infants during the first 6 months of life. Chronobiol. Int. 28, 330–337. doi: 10.3109/07420528.2011.565895. PMID: 21539424

