# Correction: Circadian system functional status and sleep in blind subjects with and without conscious light perception

**DOI:** 10.3389/fphys.2026.1858443

**Published:** 2026-04-27

**Authors:** David Martínez-Martínez, Pedro González-Romero, Beatriz Rodríguez-Morilla, María Ángeles Bonmatí-Carrión, María Ángeles Rol, Pedro Francisco Almaida-Pagán

**Affiliations:** 1 Chronobiology and Sleep Laboratory, University of Murcia, Murcia, Spain; 2 IMIB-Arrixaca, Murcia, Spain; 3 Ciber Fragilidad y Envejecimiento Saludable (CIBERFES), Instituto de Salud Carlos III, Madrid, Spain; 4 Kronohealth SL, Murcia, Spain; 5 Department of Anatomy and Psychobiology, University of Murcia, Research Institute on Ageing (IUIE), Murcia, Spain

**Keywords:** blindness, circadian photoreception, daily habits, feeding times, light exposure, sleep

In the published article there was an error in the affiliation list.

Affiliation 3 “Ciber Fragilidad y Envejecimiento Saludable (CIBERFES), Instituto de Salud Carlos III, Madrid, Spain” was omitted for authors Pedro González-Romero, Beatriz Rodríguez-Morilla, María Ángeles Rol and Pedro Francisco Almaida-Pagán. This affiliation has now been assigned for authors Pedro González-Romero, Beatriz Rodríguez-Morilla, María Ángeles Rol and Pedro Francisco Almaida-Pagán.

Affiliation 2 “IMIB-Arrixaca, Murcia, Spain” was omitted for author María Ángeles Bonmatí-Carrión. This affiliation has now been assigned for author María Ángeles Bonmatí-Carrión.

Author María Ángeles Bonmatí-Carrión was erroneously assigned to affiliation 1 “Chronobiology and Sleep Laboratory, University of Murcia, Murcia, Spain, 2IMIB-Arrixaca”. This affiliation has now been removed for author María Ángeles Bonmatí-Carrión.

The original version of this article has been updated.

