# Supplementary material for: Circadian system functional status and sleep in blind subjects with and without conscious light perception
Source: Front Physiol. 2026 Apr 13;17:1787735. doi: 10.3389/fphys.2026.1787735 (PMC13159208; doi:10.3389/fphys.2026.1787735)
Supplement: Supplementary file 1 [file Table1.docx]

| Supplementary Table 1. Non-adjusted *p* values obtained during statistical analysis and their corresponding *p*-adjusted values after applying the Benjamini-Hochber method for multiple testing. | | | | |
| --- | --- | --- | --- | --- |
| Descriptors | **Non-adjusted *p*** | **Variable** | **Comparison** | **p-adjusted** |
| M10 | 0.003 | Acceleration of Movement | Control vs Blind | 0.014 |
| M10T | 0.879 | Acceleration of Movement | Control vs Blind | 0.905 |
| L5 | 0.169 | Acceleration of Movement | Control vs Blind | 0.261 |
| L5T | 0.840 | Acceleration of Movement | Control vs Blind | 0.905 |
| IS | 0.889 | Acceleration of Movement | Control vs Blind | 0.905 |
| IV | 0.181 | Acceleration of Movement | Control vs Blind | 0.261 |
| NRA | 0.002 | Acceleration of Movement | Control vs Blind | 0.010 |
| ES | 0.905 | Acceleration of Movement | Control vs Blind | 0.905 |
| CHS | 0.006 | Acceleration of Movement | Control vs Blind | 0.018 |
| Physical Activity Vigorous | 0.033 | Acceleration of Movement | Control vs Blind | 0.072 |
| Physical Activity Moderate | 0.001 | Acceleration of Movement | Control vs Blind | 0.010 |
| Physical Activity Light | 0.113 | Acceleration of Movement | Control vs Blind | 0.210 |
| Physical Activity Sedentary | 0.007 | Acceleration of Movement | Control vs Blind | 0.018 |
| M5 | 0.006 | ANOVA Gender Temp | Control vs Blind | 0.030 |
| M5T | 0.210 | ANOVA Gender Temp | Control vs Blind | 0.336 |
| L10 | 0.417 | ANOVA Gender Temp | Control vs Blind | 0.501 |
| L10T | 0.844 | ANOVA Gender Temp | Control vs Blind | 0.844 |
| IS | 0.012 | ANOVA Gender Temp | Control vs Blind | 0.030 |
| IV |  | ANOVA Gender Temp | Control vs Blind |  |
| NRA | 0.015 | ANOVA Gender Temp | Control vs Blind | 0.030 |
| ES | 0.438 | ANOVA Gender Temp | Control vs Blind | 0.501 |
| CHS | 0.015 | ANOVA Gender Temp | Control vs Blind | 0.030 |
| M10 | 0.016 | BlueLight Exposure | Control vs Blind | 0.144 |
| M10T | 0.644 | BlueLight Exposure | Control vs Blind | 0.924 |
| L5 | 0.924 | BlueLight Exposure | Control vs Blind | 0.924 |
| L5T | 0.395 | BlueLight Exposure | Control vs Blind | 0.862 |
| IS | 0.067 | BlueLight Exposure | Control vs Blind | 0.201 |
| IV | 0.868 | BlueLight Exposure | Control vs Blind | 0.924 |
| NRA | 0.785 | BlueLight Exposure | Control vs Blind | 0.924 |
| ES | 0.479 | BlueLight Exposure | Control vs Blind | 0.862 |
| CHS | 0.040 | BlueLight Exposure | Control vs Blind | 0.180 |
| Fasting Duration | 0.029 | Fasting Variables | Control vs Blind | 0.037 |
| Dinner Time | 0.019 | Fasting Variables | Control vs Blind | 0.034 |
| CPDdinner | 0.016 | Fasting Variables | Control vs Blind | 0.034 |
| TDSO | 0.058 | Fasting Variables | Control vs Blind | 0.058 |
| TDMS | 0.020 | Fasting Variables | Control vs Blind | 0.034 |
| M10 | 0.010 | Light Exposure | Control vs Blind | 0.038 |
| M10T | 0.962 | Light Exposure | Control vs Blind | 0.962 |
| L5 | 0.548 | Light Exposure | Control vs Blind | 0.669 |
| L5T | 0.343 | Light Exposure | Control vs Blind | 0.539 |
| IS | 0.041 | Light Exposure | Control vs Blind | 0.075 |
| IV | 0.740 | Light Exposure | Control vs Blind | 0.814 |
| NRA | 0.009 | Light Exposure | Control vs Blind | 0.038 |
| ES | 0.502 | Light Exposure | Control vs Blind | 0.669 |
| CHS | 0.025 | Light Exposure | Control vs Blind | 0.055 |
| Light Indoors | 0.004 | Light Exposure | Control vs Blind | 0.038 |
| Light Outdoors | 0.022 | Light Exposure | Control vs Blind | 0.055 |
| M5 | 0.002 | Sleep Circadian | Control vs Blind | 0.018 |
| M5T | 0.483 | Sleep Circadian | Control vs Blind | 0.621 |
| L10 | 0.422 | Sleep Circadian | Control vs Blind | 0.621 |
| L10T | 0.772 | Sleep Circadian | Control vs Blind | 0.772 |
| IS | 0.030 | Sleep Circadian | Control vs Blind | 0.068 |
| IV | 0.443 | Sleep Circadian | Control vs Blind | 0.621 |
| NRA | 0.007 | Sleep Circadian | Control vs Blind | 0.030 |
| ES | 0.606 | Sleep Circadian | Control vs Blind | 0.682 |
| CHS | 0.017 | Sleep Circadian | Control vs Blind | 0.051 |
| Sleep Latency | 0.224 | Sleep Parameters | Control vs Blind | 0.532 |
| Sleep Interval | 0.651 | Sleep Parameters | Control vs Blind | 0.883 |
| WASO | 0.134 | Sleep Parameters | Control vs Blind | 0.363 |
| Sleep Efficiency | 0.012 | Sleep Parameters | Control vs Blind | 0.046 |
| Awakenings (nº/h) | 0.278 | Sleep Parameters | Control vs Blind | 0.586 |
| Total time of movement | 0.997 | Sleep Parameters | Control vs Blind | 0.997 |
| WT during sleep | 0.815 | Sleep Parameters | Control vs Blind | 0.902 |
| Visible light during sleep | 0.510 | Sleep Parameters | Control vs Blind | 0.807 |
| Blue light during sleep | 0.386 | Sleep Parameters | Control vs Blind | 0.671 |
| Activity 2h before SO | 0.854 | Sleep Parameters | Control vs Blind | 0.902 |
| Visible light 2h before SO | 0.040 | Sleep Parameters | Control vs Blind | 0.125 |
| Blue light 2h before SO | 0.725 | Sleep Parameters | Control vs Blind | 0.902 |
| WT 2h after waking | 0.798 | Sleep Parameters | Control vs Blind | 0.902 |
| Activity 2h after waking | 0.001 | Sleep Parameters | Control vs Blind | 0.006 |
| Visible light 2h after waking | 0.000 | Sleep Parameters | Control vs Blind | 0.000 |
| Blue light 2h after waking | 0.003 | Sleep Parameters | Control vs Blind | 0.021 |
| Napping time | 0.553 | Sleep Parameters | Control vs Blind | 0.808 |
| A/T index | 0.005 | Sleep Parameters | Control vs Blind | 0.024 |
| Internal Syncronization | 0.389 | Sleep Parameters | Control vs Blind | 0.671 |
| M10 | 0.083 | TAPL | Control vs Blind | 0.150 |
| M10T | 0.855 | TAPL | Control vs Blind | 0.909 |
| L5 | 0.023 | TAPL | Control vs Blind | 0.054 |
| L5T | 0.909 | TAPL | Control vs Blind | 0.909 |
| IS | 0.024 | TAPL | Control vs Blind | 0.054 |
| IV | 0.130 | TAPL | Control vs Blind | 0.196 |
| NRA | 0.010 | TAPL | Control vs Blind | 0.047 |
| ES | 0.549 | TAPL | Control vs Blind | 0.706 |
| CHS | 0.009 | TAPL | Control vs Blind | 0.047 |
| M5 | 0.627 | Temperature | Control vs Blind | 0.788 |
| M5T | 0.396 | Temperature | Control vs Blind | 0.788 |
| L10 | 0.689 | Temperature | Control vs Blind | 0.788 |
| L10T | 0.528 | Temperature | Control vs Blind | 0.788 |
| IS | 0.406 | Temperature | Control vs Blind | 0.788 |
| IV |  | Temperature | Control vs Blind |  |
| NRA | 0.934 | Temperature | Control vs Blind | 0.934 |
| ES | 0.399 | Temperature | Control vs Blind | 0.788 |
| CHS | 0.546 | Temperature | Control vs Blind | 0.788 |
| M10 | 0.792 | Time in Movement | Control vs Blind | 0.985 |
| M10T | 0.602 | Time in Movement | Control vs Blind | 0.985 |
| L5 | 0.043 | Time in Movement | Control vs Blind | 0.383 |
| L5T | 0.985 | Time in Movement | Control vs Blind | 0.985 |
| IS | 0.964 | Time in Movement | Control vs Blind | 0.985 |
| IV | 0.950 | Time in Movement | Control vs Blind | 0.985 |
| NRA | 0.521 | Time in Movement | Control vs Blind | 0.985 |
| ES | 0.775 | Time in Movement | Control vs Blind | 0.985 |
| CHS | 0.109 | Time in Movement | Control vs Blind | 0.493 |
| M10 | 0.212 | Acceleration of Movement | CLP vs NCLP | 0.637 |
| M10T | 0.593 | Acceleration of Movement | CLP vs NCLP | 0.873 |
| L5 | 0.782 | Acceleration of Movement | CLP vs NCLP | 0.873 |
| L5T | 0.550 | Acceleration of Movement | CLP vs NCLP | 0.873 |
| IS | 0.873 | Acceleration of Movement | CLP vs NCLP | 0.873 |
| IV | 0.182 | Acceleration of Movement | CLP vs NCLP | 0.637 |
| NRA | 0.165 | Acceleration of Movement | CLP vs NCLP | 0.637 |
| ES | 0.863 | Acceleration of Movement | CLP vs NCLP | 0.873 |
| CHS | 0.661 | Acceleration of Movement | CLP vs NCLP | 0.873 |
| M10 | 0.534 | BlueLight Exposure | CLP vs NCLP | 0.923 |
| M10T | 0.710 | BlueLight Exposure | CLP vs NCLP | 0.923 |
| L5 | 0.195 | BlueLight Exposure | CLP vs NCLP | 0.923 |
| L5T | 0.380 | BlueLight Exposure | CLP vs NCLP | 0.923 |
| IS | 0.814 | BlueLight Exposure | CLP vs NCLP | 0.923 |
| IV | 0.820 | BlueLight Exposure | CLP vs NCLP | 0.923 |
| NRA | 0.234 | BlueLight Exposure | CLP vs NCLP | 0.923 |
| ES | 0.570 | BlueLight Exposure | CLP vs NCLP | 0.923 |
| CHS | 0.952 | BlueLight Exposure | CLP vs NCLP | 0.952 |
| M10 | 0.432 | Light Exposure | CLP vs NCLP | 0.767 |
| M10T | 0.682 | Light Exposure | CLP vs NCLP | 0.767 |
| L5 | 0.606 | Light Exposure | CLP vs NCLP | 0.767 |
| L5T | 0.490 | Light Exposure | CLP vs NCLP | 0.767 |
| IS | 0.689 | Light Exposure | CLP vs NCLP | 0.767 |
| IV | 0.742 | Light Exposure | CLP vs NCLP | 0.767 |
| NRA | 0.413 | Light Exposure | CLP vs NCLP | 0.767 |
| ES | 0.514 | Light Exposure | CLP vs NCLP | 0.767 |
| CHS | 0.767 | Light Exposure | CLP vs NCLP | 0.767 |
| M10 | 0.684 | Sleep Circadian | CLP vs NCLP | 0.967 |
| M10T | 0.727 | Sleep Circadian | CLP vs NCLP | 0.967 |
| L5 | 0.363 | Sleep Circadian | CLP vs NCLP | 0.967 |
| L5T | 0.860 | Sleep Circadian | CLP vs NCLP | 0.967 |
| IS | 0.789 | Sleep Circadian | CLP vs NCLP | 0.967 |
| IV | 0.100 | Sleep Circadian | CLP vs NCLP | 0.899 |
| NRA | 0.855 | Sleep Circadian | CLP vs NCLP | 0.967 |
| ES | 0.980 | Sleep Circadian | CLP vs NCLP | 0.980 |
| CHS | 0.763 | Sleep Circadian | CLP vs NCLP | 0.967 |
| Sleep Latency | 0.475 | Sleep Parameters | CLP vs NCLP | 0.881 |
| Sleep Interval | 0.288 | Sleep Parameters | CLP vs NCLP | 0.881 |
| WASO | 0.778 | Sleep Parameters | CLP vs NCLP | 0.881 |
| Sleep Efficiency | 0.706 | Sleep Parameters | CLP vs NCLP | 0.881 |
| Awakenings (nº/h) | 0.016 | Sleep Parameters | CLP vs NCLP | 0.197 |
| Total time of movement | 0.629 | Sleep Parameters | CLP vs NCLP | 0.881 |
| WT during sleep | 0.979 | Sleep Parameters | CLP vs NCLP | 0.979 |
| Visible light during sleep | 0.788 | Sleep Parameters | CLP vs NCLP | 0.881 |
| Blue light during sleep | 0.779 | Sleep Parameters | CLP vs NCLP | 0.881 |
| Activity 2h before SO | 0.375 | Sleep Parameters | CLP vs NCLP | 0.881 |
| Visible light 2h before SO | 0.108 | Sleep Parameters | CLP vs NCLP | 0.514 |
| Blue light 2h before SO | 0.031 | Sleep Parameters | CLP vs NCLP | 0.197 |
| WT 2h after waking | 0.644 | Sleep Parameters | CLP vs NCLP | 0.881 |
| Activity 2h after waking | 0.304 | Sleep Parameters | CLP vs NCLP | 0.881 |
| Visible light 2h after waking | 0.386 | Sleep Parameters | CLP vs NCLP | 0.881 |
| Blue light 2h after waking | 0.728 | Sleep Parameters | CLP vs NCLP | 0.881 |
| Napping time | 0.473 | Sleep Parameters | CLP vs NCLP | 0.881 |
| A/T index | 0.860 | Sleep Parameters | CLP vs NCLP | 0.908 |
| Internal Syncronization | 0.023 | Sleep Parameters | CLP vs NCLP | 0.197 |
| M10 | 0.683 | TAPL | CLP vs NCLP | 0.887 |
| M10T | 0.485 | TAPL | CLP vs NCLP | 0.887 |
| L5 | 0.887 | TAPL | CLP vs NCLP | 0.887 |
| L5T | 0.448 | TAPL | CLP vs NCLP | 0.887 |
| IS | 0.737 | TAPL | CLP vs NCLP | 0.887 |
| IV | 0.013 | TAPL | CLP vs NCLP | 0.120 |
| NRA | 0.593 | TAPL | CLP vs NCLP | 0.887 |
| ES | 0.570 | TAPL | CLP vs NCLP | 0.887 |
| CHS | 0.850 | TAPL | CLP vs NCLP | 0.887 |
| M10 | 0.881 | Temperature | CLP vs NCLP | 0.950 |
| M10T | 0.044 | Temperature | CLP vs NCLP | 0.269 |
| L5 | 0.549 | Temperature | CLP vs NCLP | 0.950 |
| L5T | 0.353 | Temperature | CLP vs NCLP | 0.942 |
| IS | 0.950 | Temperature | CLP vs NCLP | 0.950 |
| IV |  | Temperature | CLP vs NCLP |  |
| NRA | 0.694 | Temperature | CLP vs NCLP | 0.950 |
| ES | 0.067 | Temperature | CLP vs NCLP | 0.269 |
| CHS | 0.723 | Temperature | CLP vs NCLP | 0.950 |
| M10 | 0.317 | Time in Movement | CLP vs NCLP | 0.960 |
| M10T | 0.621 | Time in Movement | CLP vs NCLP | 0.960 |
| L5 | 0.850 | Time in Movement | CLP vs NCLP | 0.960 |
| L5T | 0.701 | Time in Movement | CLP vs NCLP | 0.960 |
| IS | 0.405 | Time in Movement | CLP vs NCLP | 0.960 |
| IV | 0.913 | Time in Movement | CLP vs NCLP | 0.960 |
| NRA | 0.297 | Time in Movement | CLP vs NCLP | 0.960 |
| ES | 0.960 | Time in Movement | CLP vs NCLP | 0.960 |
| CHS | 0.928 | Time in Movement | CLP vs NCLP | 0.960 |
