# Supplementary material for: Circadian system functional status and sleep in blind subjects with and without conscious light perception
Source: Front Physiol. 2026 Apr 13;17:1787735. doi: 10.3389/fphys.2026.1787735 (PMC13159208; doi:10.3389/fphys.2026.1787735)
Supplement: Supplementary file 3 [file Table3.docx]

Supplementary Table 3A. Time spent (in hours) in physical activities of different level of intensity from blind participants and controls.

| Physical activity (h) | Blind | Control |
| --- | --- | --- |
| Vigorous | 0.04±0.02 | 0.12±0.04 |
| **Moderate** | **0.56±0.11** | **1.18±0.12** |
| Light | 2.62±0.32 | 3.06±0.20 |
| **Sedentary** | **14.38±0.41** | **13.12±0.30** |

Data expressed as mean ± SEM (n=18 for blind and n= 26 for controls). A student’s t-test was used to compare blind participants and Control means for each parameter. Differences with a value of *p < 0.05* are shown in bold type. A two-way ANOVA was conducted to analyse possible interactions between group (blind or control) and gender. No significant differences were found (*p ≥0.05*).

Supplementary Table 3B. Time spent (in hours) in physical activities of different level of intensity from blind participants who reported to keep conscious light perception (CLP) and those who reported to lack conscious light perception (NO CLP).

| Physical activity (h) | CLP | NO CLP |
| --- | --- | --- |
| Vigorous | 0.04±0.02 | 0.04±0.03 |
| Moderate | 0.61±0.15 | 0.51±0.18 |
| Light | 2.96±0.57 | 2.27±0.28 |
| Sedentary | 14.10±0.70 | 14.65±0.44 |

Data expressed as mean ± SEM (n=9 for CLP and n= 9 for NO CLP). A student’s t-test was used to compare CLP and NO CLP means for each parameter. A two-way ANOVA was conducted to analyse possible interactions between group (CLP or NO-CLP) and gender. No significant differences were found (*p ≥0.05*).
